# Inorganic interpretation of luminescent materials encountered by the Perseverance rover on Mars

**DOI:** 10.1126/sciadv.adm8241

**Published:** 2024-09-25

**Authors:** Eva L. Scheller, Tanja Bosak, Francis M. McCubbin, Kenneth Williford, Sandra Siljeström, Ryan S. Jakubek, Scott A. Eckley, Richard V. Morris, Sergei V. Bykov, Tanya Kizovski, Sanford Asher, Eve Berger, Dina M. Bower, Emily L. Cardarelli, Bethany L. Ehlmann, Teresa Fornaro, Allison Fox, Nikole Haney, Kevin Hand, Ryan Roppel, Sunanda Sharma, Andrew Steele, Kyle Uckert, Anastasia G. Yanchilina, Olivier Beyssac, Kenneth A. Farley, Jesper Henneke, Chris Heirwegh, David A. K. Pedersen, Yang Liu, Mariek E. Schmidt, Mark Sephton, David Shuster, Benjamin P. Weiss

**Affiliations:** ^1^ Department of Earth, Atmospheric, and Planetary Sciences, Massachusetts Institute of Technology, Cambridge, MA 02139, USA.; ^2^ Astromaterials Research and Exploration Science Division, NASA Johnson Space Center, Houston, TX 77058, USA.; ^3^ Blue Marble Space Institute of Science, Seattle, WA 98104, USA.; ^4^ RISE Research Institutes of Sweden, Stockholm, Sweden.; ^5^ Jacobs, NASA Johnson Space Center, Houston, TX 77058, USA.; ^6^ Department of Chemistry, University of Pittsburgh, Pittsburgh, PA 15260, USA.; ^7^ Department of Earth Sciences, Brock University, St. Catharines, ON, Canada.; ^8^ Department of Astronomy, University of Maryland, College Park, MD 20742, USA.; ^9^ Department of Earth, Planetary and Space Sciences, University of California Los Angeles, Los Angeles, CA 90095, USA.; ^10^ Division of Geological and Planetary Sciences, California Institute of Technology, Pasadena, CA 91125, USA.; ^11^ Astrophysical Observatory of Arcetri, INAF, Florence, Italy.; ^12^ Jet Propulsion Laboratory, California Institute of Technology, Pasadena, CA 91011, USA.; ^13^ Earth and Planets Laboratory, Carnegie Institution for Science, Washington, DC 20015, USA.; ^14^ Impossible Sensing, LLC, St. Louis, MO 63118, USA.; ^15^ Institut de Minéralogie de Physique des Matériaux et de Cosmochimie, CNRS, Sorbonne Université, Muséum National d’Histoire Naturelle, Paris, France.; ^16^ Danish Technical University, Lyngby, Denmark.; ^17^ Department of Earth Science and Engineering, Imperial College London, London, UK.; ^18^ Department of Earth and Planetary Science, University of California Berkeley, Berkeley, CA 94720, USA.

## Abstract

A major objective of the Mars 2020 mission is to sample rocks in Jezero crater that may preserve organic matter for later return to Earth. Using an ultraviolet Raman and luminescence spectrometer, the Perseverance rover detected luminescence signals with maximal intensities at 330 to 350 nanometers and 270 to 290 nanometers that were initially reported as consistent with organics. Here, we test the alternative hypothesis that the 330- to 350-nanometer and 270- to 290-nanometer luminescence signals trace Ce^3+^ in phosphate and silicate defects, respectively. By comparing the distributions of luminescence signals with the rover detections of x-ray fluorescence from P_2_O_5_ and Si-bearing materials, we show that, while an organic origin is not excluded, the observed luminescence can be explained by purely inorganic materials. These findings highlight the importance of eventual laboratory analyses to detect and characterize organic compounds in the returned samples.

## Teaser

Luminescence previously attributed to organic compounds in Jezero crater rocks may instead be from inorganic sources.

## INTRODUCTION

The Perseverance rover has collected rock and soil samples in Jezero crater, Mars, for return to Earth (*1*). Future laboratory analyses of these samples will search for evidence of ancient life and prebiotic chemistry and characterize some of the oldest potentially habitable environments in the solar system (*1*–*3*). As such, a central objective of the Mars 2020 mission is to identify and acquire samples that contain potential biosignatures including possible organic matter (*1*).

Perseverance can detect organics using the Scanning Habitable Environments with Raman and Luminescence for Organics and Chemicals (SHERLOC) instrument, a deep-ultraviolet (UV) luminescence and Raman spectrometer (*4*–*7*). SHERLOC detects luminescence in the 250- to 355-nm wavelength range and UV Raman scattering over the 800 to 4500 cm^−1^ wave number range (*4*). Potential organic matter detections by the rover are based primarily on UV luminescence, which has a higher sensitivity to specifically aromatic organics than Raman scattering (*4*, *7*). In the past, these detections were typically reported as UV fluorescence (*4*–*9*). Here, we adopt the more general term “luminescence” throughout for the same observed phenomena. Luminescence is a general term for emission related to energy changes in a molecule, whereas fluorescence is the subcategory of short decay time photoluminescence.

Raman scattering can detect a broader range of different organic and inorganic compounds with higher specificity but lower sensitivity (*4*–*7*). Expected SHERLOC measurable Raman bands from macromolecular organic matter in rocks include the D- and G-bands centered at ~1350 and ~1600 cm^−1^, respectively [e.g., (*4*, *7*, *10*–*12*)]. Several other organic Raman bands and biosignature luminescence were proposed to be detectable with the SHERLOC instrument during its development (*4*, *5*). However, UV Raman bands detected on Mars by SHERLOC so far have primarily been those of inorganic salt minerals rather than organics, such as sulfate, carbonate, phosphate, perchlorate, and certain silicates, such as olivine (*8*, *13*, *14*). Sharma *et al.* (*9*) report organic Raman bands detected on Mars to date. This study also compares SHERLOC signals to observations made by the Planetary Instrument for X-ray Lithochemistry (PIXL) using x-ray fluorescence signals for measuring elemental compositions (*15*).

**Fig. 1.**
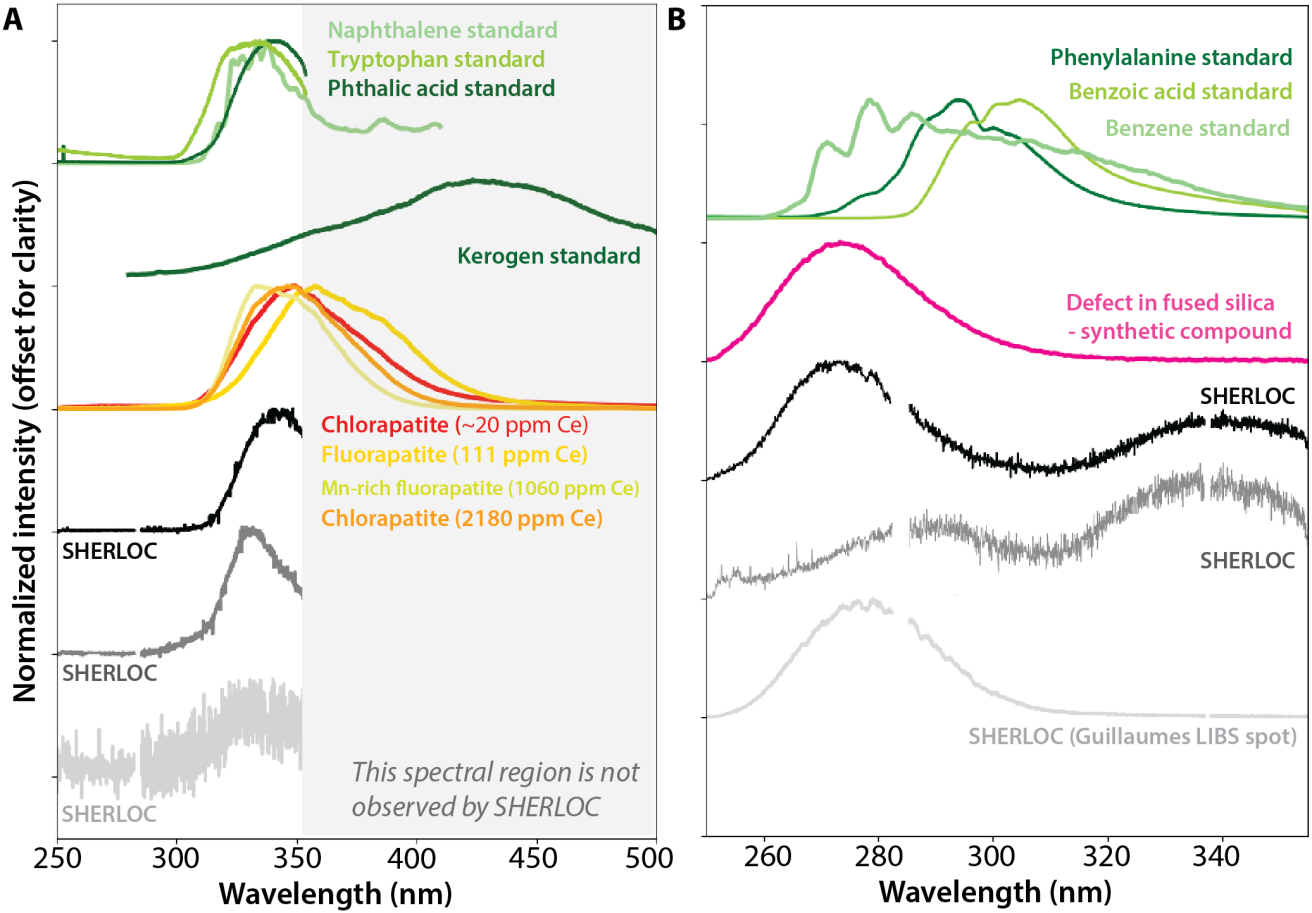
Typical SHERLOC luminescence spectra from targets in the Jezero crater floor compared to standards. (**A**) Examples of the 330- to 350-nm luminescence. Normalized SHERLOC spectra of 340-nm luminescence and 330-nm luminescence are shown in black and gray, respectively. Normalized laboratory spectra are shown in color: light to medium green denotes pure naphthalene (double ring aromatic), tryptophan (aromatic amino acid), and phthalic acid (aromatic dicarboxylic acid) [from (*7*)], dark green denotes terrestrial kerogen [from (*41*)], red denotes chlorapatite from meteorite NWA 10922 (Fig. 4), and yellow to orange shades denote natural apatite with 100 to 2200 ppm Ce from (*8*). The luminescence band intensity increases with Ce concentration (*8*). (**B**) Examples of the 270- to 290-nm luminescence. Black spectrum shows typical strong 270-nm luminescence, low in position for organic compounds, co-occurring with 340-nm luminescence. Dark gray spectrum shows 290-nm luminescence co-occurring with 340-nm luminescence. Light gray spectrum shows 280-nm luminescence from SuperCam LIBS spots in the Guillaumes rock target. Normalized laboratory spectra are shown in color: green line shows pure benzene, benzoic acid, and phenylalanine (aromatic amino acid) [from (*7*)], and pink shows spectra from oxygen vacancies in fused silica [first described in (*42*)]. Gaps in SHERLOC spectra represent detector overlap in two readout regions (*4*).

During its first year on Mars’ surface, Perseverance investigated and collected aqueously altered basaltic and olivine cumulate rocks from two informally named igneous formations (fms) on the Jezero crater floor, the Máaz and Séítah fms (*16*–*20*) (table S1). During its second year of operation, the rover investigated and collected samples from the lowest exposed beds of a sedimentary fan (*21*, *22*) (table S1). Notably, of 2400 analyzed points, SHERLOC has not reported any Raman signatures of macromolecular carbon to date except for a singular low-intensity Raman feature at ~1600 cm^−1^ in one point (*9*, *13*). On the other hand, SHERLOC identified four groups (*9*) of luminescence signals in aqueously altered igneous rocks from the crater floor that were tentatively interpreted as organic signals (*8*, *9*) (table S1). For the purposes of this study, we redefine these original four groups as three luminescence types: (1) single intensity peaks at 330 to 350 nm, (2) single intensity peaks at 270 to 290 nm, and (3) co-occurring intensity peaks at 303 and 325 nm (*20*). This study focuses on luminescence types 1 and 2 (Fig. 1). Type 3 signals, which are associated with sulfate minerals, are addressed elsewhere (*14*, *21*).

As discussed in (*8*, *9*), the 250- to 355-nm luminescence features in types 1 and 2 are consistent with, but not uniquely attributable to, the emission by one-ring or two-ring aromatic compounds. Their wavelength range overlaps with that of small aromatic and polycyclic aromatic hydrocarbons (PAHs) such as benzene and naphthalene (Fig. 1) (*7*–*9*), which are common extraterrestrial materials. For example, larger multiring PAHs in the carbonaceous chondrite Orgueil and in samples from the carbonaceous asteroid Ryugu occur at abundances ranging from <100 to 50,000 parts per million (ppm) (*23*). These abundances would likely be above SHERLOC’s luminescence detection limits for one- and two-ring aromatic compounds (*4*).

The proposed fluorescence detection of one-ring and two-ring aromatic compounds in Jezero crater is surprising for several reasons. First, three-ring and four-ring compounds (e.g., anthracene, phenathrene, and fluoranthene, and their alkylated or nitrogen-containing derivatives) are the most abundant PAHs in carbonaceous chondrites [e.g., (*23*)], asteroids (*23*), comets (*24*, *25*), and interplanetary dust particles (*26*, *27*). Substituted benzenes and naphthalene are present in some but not all of these materials [e.g., (*23*–*26*)], and some are tentatively interpreted as pyrolysis products of the macromolecular organic material or contaminants in the capturing materials [e.g., (*24*)]. Second, as mentioned above, SHERLOC Raman has not unambiguously detected organic macromolecular carbon (e.g., kerogen) in the rocks exhibiting luminescence analyzed to date, although such compounds are the most common form of organic matter in geological materials on Earth (*28*–*30*) and in extraterrestrial materials (*31*, *32*), including in martian meteorites (*12*) and in Gale Crater, Mars, as detected by the Sample Analysis at Mars instrument on the Curiosity rover (*33*, *34*). When devolatilized for mass spectrometric analysis in the laboratory, this insoluble organic material can release one-ring and two-ring aromatic compounds together with a diversity of other functional groups [e.g., (*35*–*38*)]. However, because analyses of Jezero rocks with Raman and luminescence spectroscopy do not include devolatilization or other extraction steps, they would be expected to primarily detect macromolecular carbon.

Here, we consider the alternative inorganic hypothesis: The UV luminescence signals detected by SHERLOC originate from Ce^3+^ in some minerals and defects in silica/silicates (*8*, *9*, *39*–*42*) (Fig. 1). We begin by outlining the expected spectral signals that can be produced by organic or inorganic sources detected by SHERLOC and PIXL. Then, we compare the spatial distributions of luminescence signals detected by SHERLOC with the spatial distributions of hypothesized inorganic sources of these signals measured by SHERLOC and PIXL. We study six abraded patches of igneous rocks from the Máaz fm (names: Bellegarde, Alfalfa, and Montpezat) and Séítah fm (names: Garde, Dourbes, and Quartier) and two abraded patches from the lower sedimentary beds (names: Thornton Gap and Berry Hollow). These analyses show that we cannot exclude an inorganic origin of most or all of the luminescence signals detected by the rover.

## PREDICTIONS OF ORGANIC AND INORGANIC HYPOTHESES

### Type 1 signals

Macromolecular carbon, whether of biological or abiotic origin, luminesces with a maximum around ~430 nm (*41*, *43*, *44*). Given that larger three- to five-ring PAHs also luminesce with maxima in the 400- to 500-nm range (*7*, *45*, *46*), mixtures of larger PAHs and macromolecular organics would be expected to luminesce with maxima above 400 nm. Because SHERLOC’s wavelength range is between 250 and 355 nm (Fig. 1A), it cannot fully detect this ~430-nm luminescence but could detect the part of the signal as a gentle positive slope between 300 and 355 nm if the organic sources are present in sufficiently high concentrations [described in (*41*, *45*, *46*)]. Furthermore, Raman G-bands centered at ~1600 cm^−1^ are also characteristic of macromolecular organic compounds and have been detected by SHERLOC-type UV Raman spectroscopy in terrestrial samples containing 0.01 to 0.1 wt % of bulk total organic carbon (*10*, *41*, *47*) and in meteorites (*4*, *48*). The SHERLOC meteorite calibration target, Sayh al Uhaymir (SaU) 008, contains organic compounds with resolvable Raman G-bands that spatially correlate with luminescence exhibiting a gentle positive slope characteristic of macromolecular carbon (fig. S1). As such, it is noteworthy that SHERLOC has not reported Raman G-bands in the luminescing rocks from Jezero analyzed in this study, except for a singular Raman feature mentioned above (*9*, *13*).

This leads us to consider alternative, inorganic sources of the SHERLOC luminescence signals. In particular, ppm-level Ce substituted in minor quantities in natural or synthetic minerals can emit UV luminescence when measured in the laboratory (*8*, *39*, *40*, *49*, *50*) (Fig. 1). Trace amounts of Ce^3+^ substituted into minerals emit 330- to 350-nm luminescence (*8*, *39*, *40*, *49*, *50*). Among all minerals, phosphates such as apatite and merrillite most commonly contain Ce^3+^ due to favorable substitution of lanthanides into Ca^2+^ sites. Igneous phosphates on Mars and Earth have hundreds to thousands of ppm Ce [e.g., (*51*–*53*)], whereas aqueously precipitated phosphates on Earth typically contain tens to hundreds of ppm Ce (*54*, *55*). Other geologically relevant minerals such as sulfate, carbonate, zircon, and silicates sometimes can contain ppm levels of Ce with essentially identical Ce^3+^ UV luminescence signals to those in phosphates [reviewed in (*39*)]. These minerals incorporate Ce less commonly than phosphates, which are typically the main Ce carriers in geological materials.

We first discuss the rover payload’s sensitivity to Ce. In addition to SHERLOC, we consider measurements from PIXL. While SHERLOC can detect Ce^3+^ luminescence (*8*), PIXL can detect the Ce element itself. A SHERLOC laboratory analog can detect strong luminescence from 100 ppm phosphate-hosted Ce in a single crystal (*8*) (yellow line; Fig. 1). Here, we find that 20 ppm Ce in the bulk materials of the Northwest Africa (NWA) 10922 meteorite is detectable with a SHERLOC laboratory analog (see the “Results: Comparing Predictions to Observations” section) (red line; Fig. 1). In comparison, PIXL’s detection limits are reported as dependent on integration time, which is typically 10 s per spot on Mars. A simulated PIXL Ce detection limit in a simulated basalt composition was calculated to be 600 to 900 ppm for a 10-s integration time (*56*). Thus, under typical measuring conditions on Mars, SHERLOC UV luminescence may be at least an order of magnitude more sensitive to Ce than PIXL. So far, the PIXL instrument has not confirmed detection of Ce in the investigated rocks (*56*), and typical natural bulk concentrations of approximately tens of ppm phosphate-hosted Ce in basalts are not anticipated to be detectable with PIXL (*56*).

**Fig. 2.**
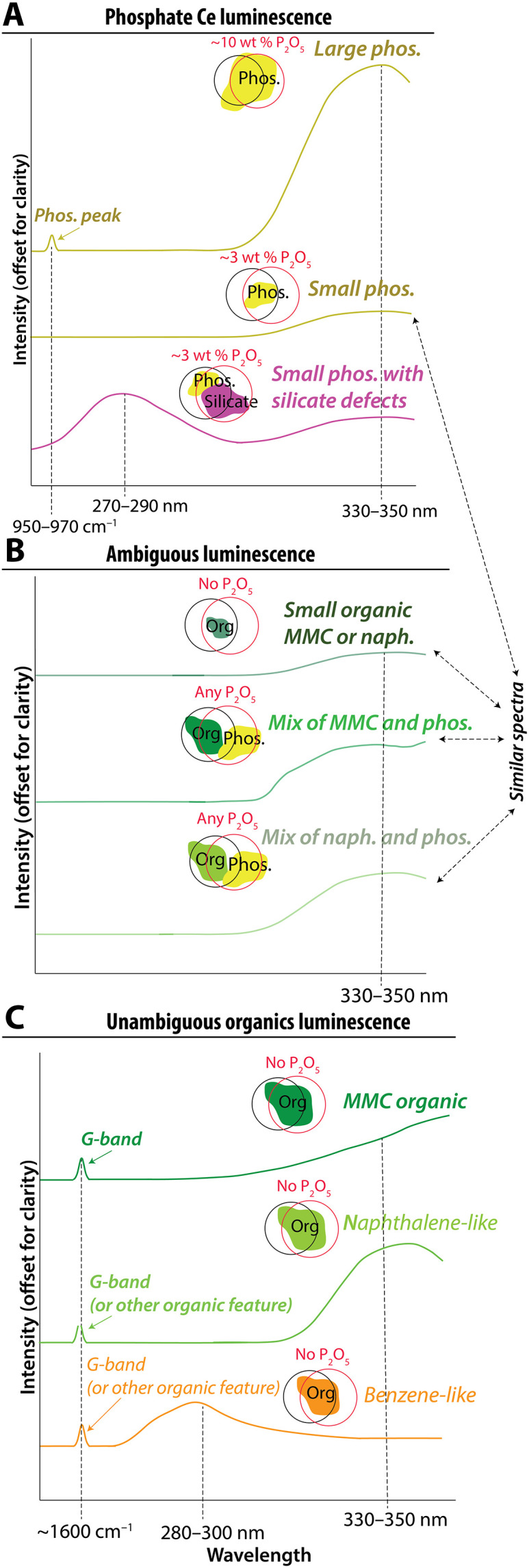
Predicted observations from inorganic, organic, and ambiguous sources of 330- to 350-nm luminescence signals. Cartoons illustrate potential sources of luminescence in the spot size of the SHERLOC laser and PIXL x-ray sources, including (**A**) inorganic sources, (**B**) ambiguous sources, and (**C**) unambiguous organic sources. These are idealized cartoon versions of spectra from Fig. 1 showing hypothesized changes in responses due to material volume covered by spot size and material mixtures. These spectra were not computed and are meant to function as hypotheses for spectral behavior. The intensity of the spectral signal can vary with material volume as represented by cartoons of phosphate minerals (yellow), other minerals (blue), and organics (green) in different sizes. Black and red circles illustrate surfaces measured by SHERLOC and PIXL, respectively; even when the two instruments target the same location on a rock, these footprints often do not perfectly overlap, such that they may sense different fractions of minerals. MMC, macromolecular carbon.

Next, we discuss the rover payload’s sensitivity to phosphate. If the luminescence at 330 to 350 nm observed by SHERLOC originates from phosphate-associated Ce, we might expect to have independent spatially correlated indicators of phosphate. In particular, SHERLOC could observe a Raman phosphate peak around 960 cm^−1^ where PIXL observes the element P, reported as the oxide P_2_O_5_ (Fig. 2). Raman bands for minerals and organics are reportedly detectable at ~0.1 wt % (*4*), depending on the material, measurement properties, and spatial distribution of the materials being analyzed. PIXL can detect P_2_O_5_ in individual x-ray points if its abundance exceeds more than ~1 wt % within the area covered by the PIXL beam diameter (0.12 mm); this sensitivity improves when many scan points are averaged together for bulk measurements (e.g., table S1). Here, we confirm that PIXL may detect up to 10 wt % phosphorus from phosphate grains even when SHERLOC does not resolve a phosphate Raman peak (see the “Results: Comparing Predictions to Observations” section).

**Fig. 3.**
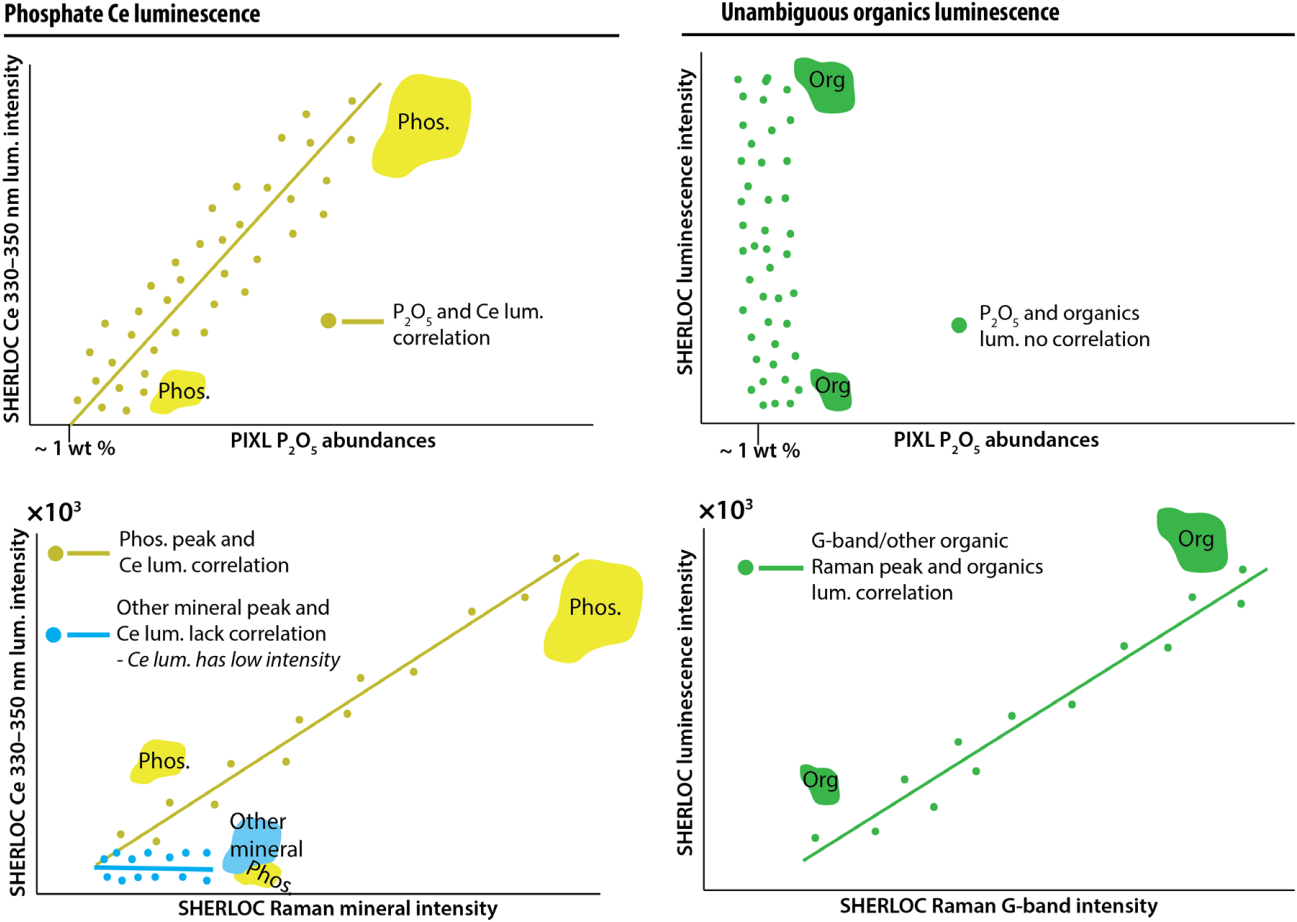
Predicted relationships between SHERLOC luminescence, PIXL detections of elements, and SHERLOC Raman detections for minerals and organics. Cartoons illustrate the anticipated relationships among signals from inorganic or organic sources. These spectra were not computed. Top row: The intensity of luminescence of Ce-bearing phosphate should correlate with the amount of PIXL-detected P_2_O_5_ (left). Likewise, if Ce is present in a mineral other than phosphate, we expect a correlation with compositional indicators of that phase (e.g., Ce-bearing sulfate luminescence should correlate with SO_3_ and defect luminescence should correlate with PIXL SiO_2_). The luminescence of organic material should not correlate with such measurements (right). These correlations could be driven by variation in the volumes of minerals within the instrument spot sizes as represented by cartoons of phosphate minerals (yellow), other minerals (blue), and organics (green) in different sizes. Bottom row: The intensity of Raman peaks of minerals that luminesce should correlate with the intensity of luminescence (left column). Unambiguous detection of organic luminescence would be accompanied by the detection of an organic Raman band (e.g., the G-band at ~1600 cm^−1^). The intensity of the organic luminescence band may correlate with the intensity of the organic Raman band due to changing volume of the organic material in the instrument spot sizes (right column).

Predictions of the inorganic hypothesis described above differ from the expected observations due to organic sources of luminescence at 330 to 350 nm. Unambiguous determination of organic matter would combine detection of UV luminescence together with a G-band in Raman spectra (Fig. 2). The intensities of this luminescence and G-band may even be correlated (Fig. 3, right column). Furthermore, luminescence with a predominant organic source is not expected to exhibit a spatial correlation with phosphate SHERLOC Raman peaks and PIXL detections of P_2_O_5_ (Fig. 3, right column) unless some geologic or biologic process, such as the precipitation of organic-preserving authigenic minerals, generated such an association. Organic materials that occur in mixtures with Ce^3+^-bearing materials would lead to ambiguous interpretations of 330- to 350-nm luminescence (Fig. 2B).

**Fig. 4.**
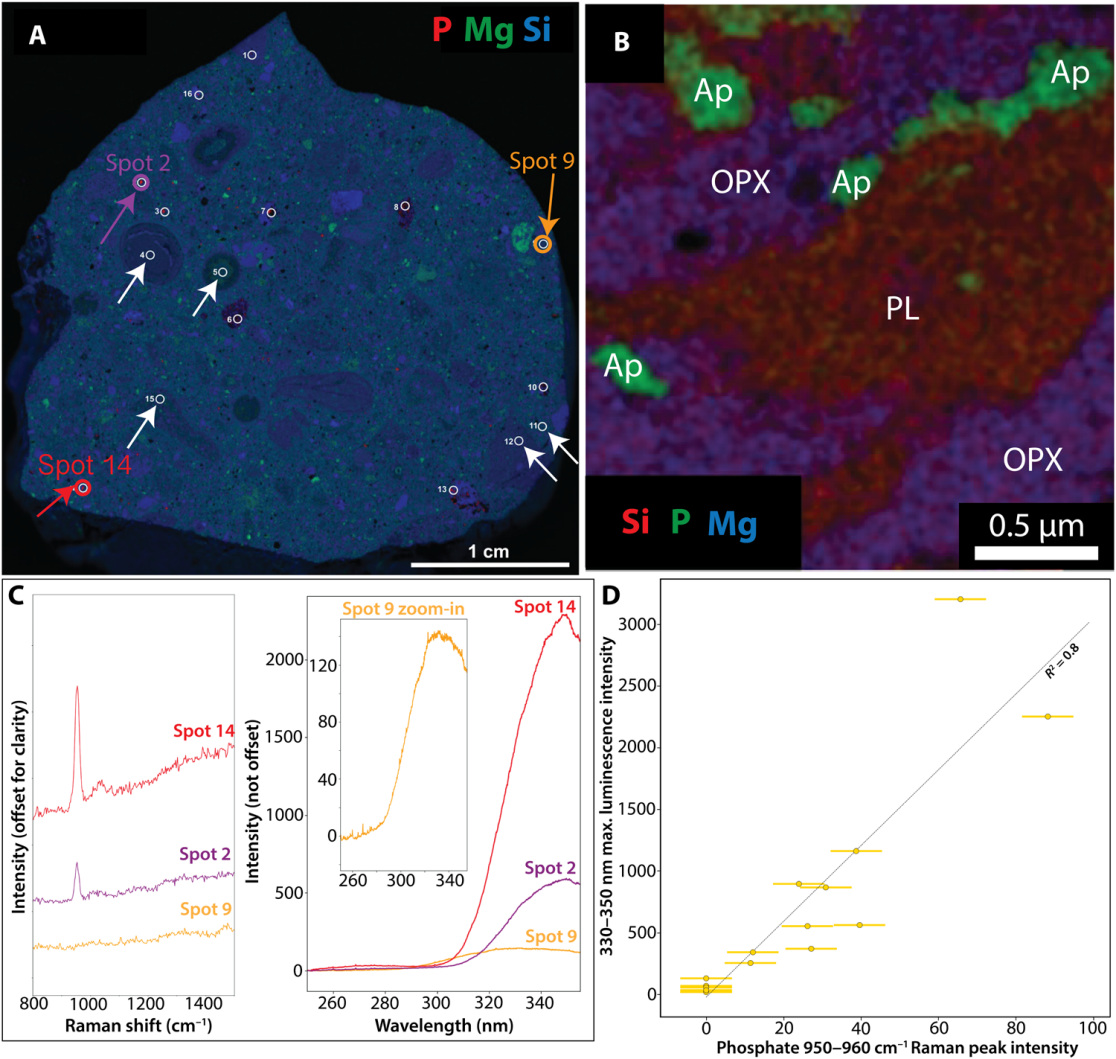
Laboratory measurements of phosphate in martian meteorite NWA 10922. (**A**) X-ray fluorescence composite map. Red color shows P abundance, green shows Mg abundance, and blue shows Si abundance. White circles outline areas analyzed by ACRONM. White arrows indicate matrix and clasts that contain nanometer-to-micrometer scale phosphate detectable by EDX-TEM only [shown in (B)]. Red, purple, and orange arrows (spots 2, 9, and 14) and circles show the location of spectra shown in (C) and (D). (**B**) Representative EDX-TEM image of nanometer-to-micrometer sized phosphates in the NWA 7034 (paired with NWA 10922) matrix (*62*). Red color shows Si abundance, green shows P abundance, and blue shows Mg abundance. (**C**) ACRONM Raman and luminescence spectra of phosphate grains (red and purple) and matrix material (orange). Note that Ce luminescence is stronger when the phosphate Raman peak is stronger and vice versa (red versus purple versus orange spectra). (**D**) Observed linear correlation between Ce luminescence and phosphate Raman peak intensities as predicted in Fig. 3. The luminescence has lower intensity when a phosphate Raman peak is weaker. Error bars are derived from the range of variation in pixel counts from scans in which no material is measured; note that luminescence error bars are smaller than symbols.

Sources of luminescence bands can also be identified by the band shapes (Fig. 2). Both inorganic and organic luminescence bands are mixtures of electron transitions with assumed Gaussian emission profiles. Experiments and theory predict a ~340-nm and ~360-nm Gaussian doublet from Ce^3+^ in the Ca(I) site in phosphate related to two well-known electron transitions [5d → ^2^F_5/2_ and ^2^F_7/2_ (4f)]. These transition bands are wide and overlapping and will therefore display as a single asymmetric band centered at 330 to 350 nm and only rarely separated such that both emission bands can be directly observed (*8*, *39*, *40*, *49*, *50*) (Figs. 1 to 4). Because SHERLOC does not observe luminescence outside of 250 to 355 nm, phosphate-associated Ce^3+^ luminescence will only be partially resolved (Figs. 1 and 2). The maximum emission centers of the Ce^3+^ luminescence doublet can readily shift down and up in wavelength depending on the coordination environment, but the predicted bands would retain the asymmetric shape composed of the two transitions (*8*, *39*, *40*, *49*, *50*). In contrast, the shape of the 330- to 350-nm luminescence bands emitted by various aromatic organics and macromolecular organic material cannot be modeled as a double Gaussian mixture in SHERLOC spectra but may potentially be modeled through more complex material mixtures [Fig. 1; (*41*, *45*, *46*)].

### Type 2 signals

The source of luminescence centered at 270 to 275 nm (type 2) is less well constrained. Some luminescence maxima observed by Perseverance are shifted toward lower wavelengths relative to all known one-ring pure aromatic organic standards (black line, Fig. 1B) [e.g., (*8*)] (dark gray line, Fig. 1B). Furthermore, naturally occurring organic compounds in rocks, such as macromolecular organic carbon, have not yet been observed to exhibit such emissions (*41*, *47*, *48*). Ce-bearing phosphate minerals also cannot account for luminescence with maxima at 270 to 290 nm. On the other hand, defects documented in silica, feldspars, and other aluminosilicates can produce a single Gaussian transition 270- to 290-nm band (*42*, *57*–*59*). Extensive studies of nongeological materials such as fused silica have shown that twofold coordinated silicon can produce 270- to 290-nm luminescence (*42*, *57*) (Fig. 1B). Defect-related 270- to 290-nm luminescence can also be observed in alkali feldspar minerals and a variety of geological aluminosilicates, where it likely arises from structural defects (*58*, *59*). SHERLOC observed such luminescence in the spots at the surface of a Jezero rock that had been analyzed by the SuperCam instrument’s LIBS laser (*8*).

The Perseverance rover payload lacks capabilities to confirm the presence of defects in silica/silicates independently of luminescence detections. If 270- to 290-nm luminescence occurs in the absence of silica/silicate, a potential organic source of luminescence could be considered more likely.

### Summary of predictions

Overall, we expect the following observations for an organic origin of luminescence signals detected by SHERLOC, listed in approximate order of importance:

1) Spatial correlation of Raman G-bands detected by SHERLOC and type 1 or type 2 luminescence.

2) No spatial correlation between phosphate detected by PIXL and type 1 luminescence.

3) No spatial correlation between phosphate detected by SHERLOC Raman and type 1 luminescence.

4) No spatial correlation between SHERLOC type 2 luminescence and silica/aluminosilicates detected by PIXL, which would directly exclude inorganic origin.

5) Any conceivable type 1 and type 2 luminescence shape and wavelength positions that are incompatible with the Gaussian doublet expected from Ce^3+^ and the single Gaussian shape expected from silica/silicate defects. Specifically, these could include the presence of vibronic bands or a positive slope from 300 to 350 nm indicative of larger PAHs and macromolecular carbon.

## RESULTS: COMPARING PREDICTIONS TO OBSERVATIONS

### SHERLOC’s ability to detect Ce luminescence and phosphate Raman signals

To test the predictions outlined in the previous section (Figs. 2 and 3), we first demonstrate that the SHERLOC laboratory analog, the Analogue Complementary Raman for Operations oN Mars (ACRONM), and a laboratory μXRF instrument can detect the luminescence from Ce-containing phosphate minerals by measuring the martian meteorite NWA 10922, a member of the NWA 7034 pairing group (*60*) (Materials and Methods). The meteorite contains 1- to 100-μm-sized grains of Cl-rich apatite with 40.5 wt % P_2_O_5_ (*61*) and 190 to 1000 ppm Ce (*62*). Its matrix has ~1 wt % P_2_O_5_ hosted by ubiquitous <1-μm-sized Cl-rich apatite grains (*62*, *63*) and has a bulk Ce abundance of about 20 ppm (*64*) (Fig. 4B). Apatite and merrillite are expected to exhibit the same ~340- and ~360-nm Ce electron transition bands, while monazite can exhibit ~320- and ~340-nm Ce electron transition bands. All three phosphate minerals have an indistinguishable PO_4_^3−^ Raman vibrational stretching mode (ν_1_) under ACRONM UV excitation (*49*, *50*).

We collected Raman and luminescence spectra from 10 phosphate grains >20 μm in size, six phosphate-containing matrix areas containing <0.1 to 0.5 μm phosphate grains, and six plagioclase and pyroxene grains in NWA10922 (Fig. 4 and figs. S2 to S4). All phosphate grains resolvable with μXRF exhibited a primary Raman symmetric PO_4_^3−^ vibrational stretching mode (ν_1_) at ~945 to 960 cm^−1^ (fig. S4) and a prominent luminescence emission at ~330 to 350 nm that was distinguishable from the luminescence of all other investigated materials in the meteorite (Fig. 4 and fig. S2). The intensities of the phosphate Raman peak and the ~330- to 350-nm luminescence were positively correlated with *R*^2^ = 0.8 (Fig. 4). The asymmetric Gaussian shape of these luminescence bands could be explained with a ~340- and ~360-nm double Gaussian mixture expected for Ce^3+^ electron transitions in the Ca(I) site of apatite [e.g., (*39*, *40*, *49*, *50*)], although solutions are nonunique (fig. S3). The phosphate grains in the matrix are much smaller than the PIXL and SHERLOC spot sizes and the resolution of μXRF maps collected in this study but were resolved by transmission electron microscopy (TEM) (*62*) (Fig. 4 and fig. S2). Analyses of plagioclase and pyroxene grains also yielded ~330- to 350-nm luminescence varying in intensity between 20 and 220 counts that exhibited a lower intensity by one to two orders of magnitude and a different shape than the luminescence from phosphate grains (fig. S2; Supplementary Text). This unanticipated 330- to 350-nm luminescence from plagioclase and pyroxene grains most likely arose from matrix beneath the plagioclase and pyroxene grains (Supplementary Text). These analyses confirm the hypotheses presented in Figs. 2 and 3 that (i) a laboratory analog of SHERLOC can detect the luminescence from Ce in martian phosphate-containing samples; (ii) the grain sizes of phosphate minerals influence the detections of phosphorus and phosphate by the laboratory analogs of PIXL and SHERLOC UV Raman; and (iii) luminescence from Ce-bearing phosphate correlates with the intensity of phosphate Raman peaks.

### Correlations of SHERLOC 330- to 350-nm luminescence with PIXL element concentrations

**Fig. 5.**
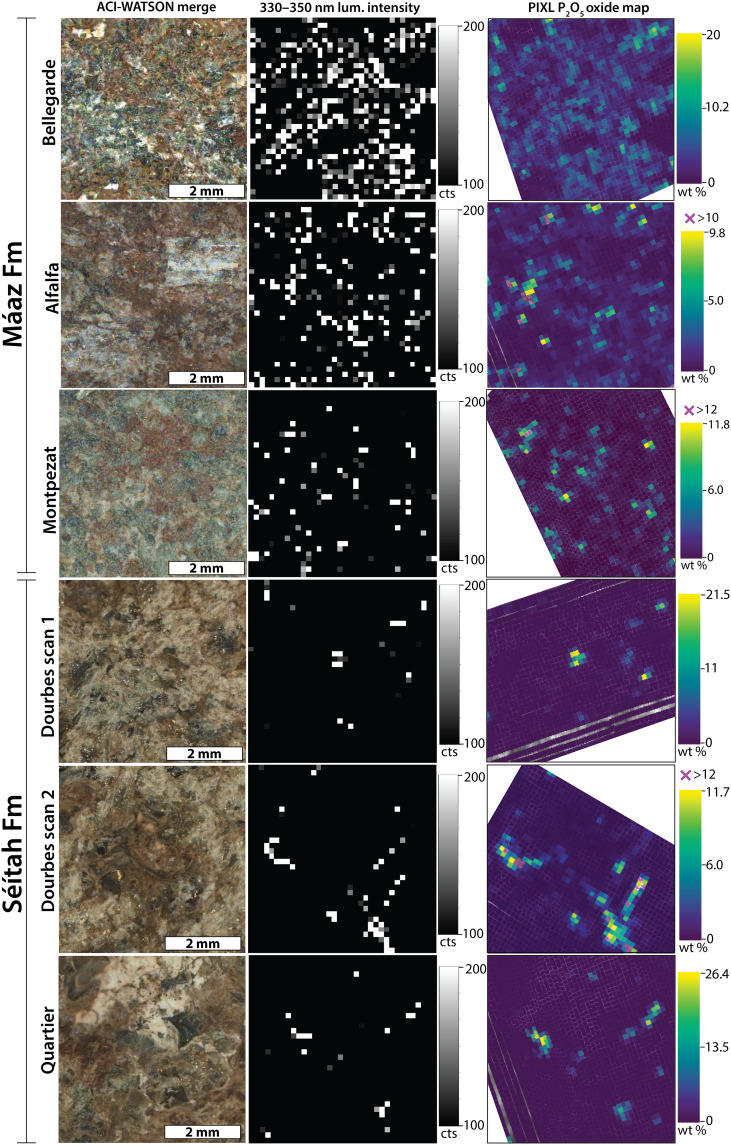
Comparisons of SHERLOC and PIXL observations from the Jezero crater floor. Left column: Colorized ACI-WATSON image merge products. Middle column: Maps of SHERLOC 330- to 350-nm luminescence intensity in counts (cts). Black color indicates no 330- to 350-nm luminescence above ~100 counts. Right column: Maps of PIXL P_2_O_5_ in wt %. Comparison of middle and right columns shows that luminescence and P_2_O_5_ hotspots correlate in rocks with both high and low P_2_O_5_ content.

**Fig. 6.**
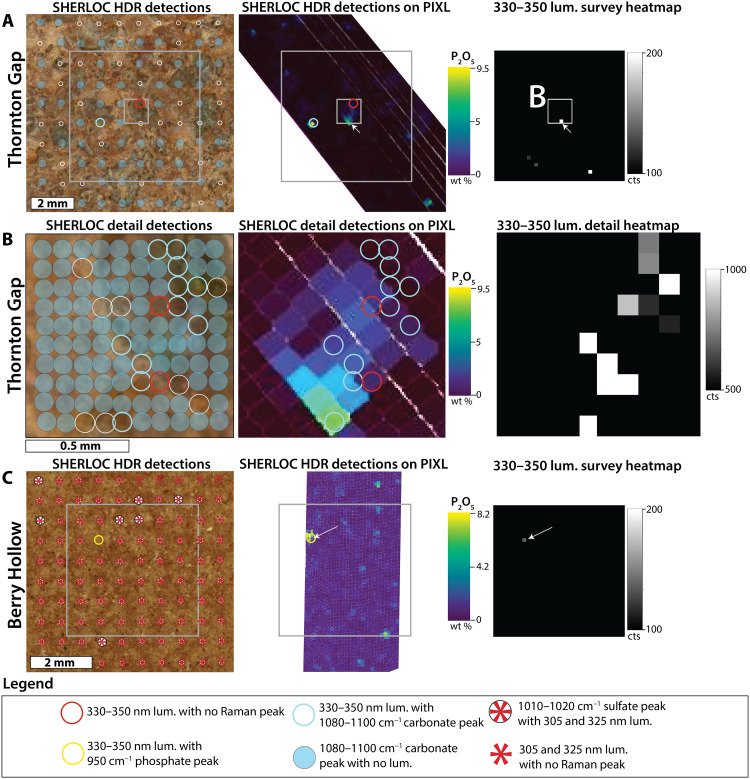
Comparisons of SHERLOC and PIXL observations from the Jezero fan front. (**A**) HDR scan of the Thornton Gap abrasion in the lower Jezero sedimentary fan. Location of the smaller SHERLOC detail scan footprint in (B) is shown in a small white square. Location of the SHERLOC survey scan in the right column is shown as the larger white square. (**B**) Data from the detail scan of the Thornton Gap abrasion. (**C**) Data from the Berry Hollow abrasion in the Jezero sedimentary fan. The left column shows scan points on the colorized ACI-WATSON image merge. Raman and luminescence detections are symbolized with colored circles and asterisks, respectively, on the colorized ACI-WATSON image merge. Open white circles indicate that no Raman features were detected. The middle column shows the 330- to 350-nm luminescence detection locations on coregistered and warped PIXL P_2_O_5_ wt % map. The right column shows the SHERLOC luminescence heatmap. Note the spatial association of both 330- and 340- to 350-nm luminescence to P_2_O_5_ hotspots (white arrow). In Thornton Gap, SHERLOC Raman detected only carbonate. Sulfate-associated 305- and 325-nm doublet luminescence in the Berry Hollow target reported in (*21*) are not examined in detail.

To further test the predictions outlined in the “Summary of predictions” section (Figs. 2 and 3), we sought to determine whether SHERLOC 330- to 350-nm luminescence exhibited statistically significant positive correlations with PIXL-detected P_2_O_5_. We analyzed three abraded patches (Bellegarde, Alfalfa, and Montpezat) in basaltic and trachyandesitic rocks from the Máaz fm (*16*), three abraded patches (Garde, Dourbes, and Quartier) in olivine cumulate rocks from the Séítah fm (*16*), and two abraded patches (Thornton Gap and Berry Hollow) from the sedimentary fan front (*21*, *22*). A detailed description of each rock target and related bulk measurements can be found in the Supplementary Materials (table S1). The 330- to 350-nm luminescence was detected in all of these rocks (Figs. 5 and 6) and was most abundant in the targets from the Máaz fm. These targets contained a higher P_2_O_5_ wt % compared to the olivine cumulate rocks from Séítah fm and sedimentary fan front (table S1). In the Séítah fm, 330- to 350-nm luminescence was associated primarily with brown microcrystalline materials interpreted as a mixture of carbonates, phosphate, and silicate material (*8*, *9*) (figs. S7 and S8). In Jezero fan sediments, 330- to 350-nm luminescence was associated with detrital sand grains (Fig. 6). Modeling the 330- to 350-nm luminescence spectral envelope with Gaussian bands in all targets with 330- to 350-nm luminescence supported a common luminescence source (fig. S12).

The distribution of luminescence features with maxima at 330 to 350 nm in survey scans visually matched the distribution of PIXL-identified P_2_O_5_. We tested the statistical robustness of these spatial associations by comparing the spatial distribution of luminescence in 36 × 36 pixel SHERLOC survey scans (with pixel spacings of 0.144 mm) to the distributions of elements in PIXL scans (with 0.125-mm spacings) (Fig. 5 and Materials and Methods) and calculating *r*, *SSIM*, and *P* values (Table 1 and table S2). To confirm that the statistical indicators for all correlations between phosphorus and luminescence could not be produced by a random dataset, we also generated a set of spatially randomized distributions of the luminescence values (figs. S10 and S11) and calculated the statistical indicators. Statistical indicators that tested the robustness of the spatial correlations between the distributions of luminescence and P_2_O_5_ (Table 1 and table S2) were consistently higher than those related to the distributions of other elements as well as stochastic datasets (Table 1, table S2, and figs. S10 and S11). In Dourbes, we observed additional positive correlations between the 330- to 350-nm luminescence and Al_2_O_3_, CaO, Na_2_O, and K_2_O, negative correlations with FeO_T_ and MgO, and no correlation with Cl. These Al_2_O_3_, CaO, Na_2_O, and K_2_O correlations were systematically weaker than that with P_2_O_5_ wt % (Table 1 and table S2). Overall, these correlations reflected the co-occurrence of P with Al, Ca, Na, and K in PIXL maps and the lack of luminescence in Fe- and Mg-rich igneous materials in Dourbes. The absence of a correlation with Cl also indicates that the luminescence is unlikely to arise from oxychlorine phases such as perchlorate, and that phosphates are not preferably Cl-bearing. In Thornton Gap and Berry Hollow, PIXL x-ray fluorescence measured between 1 and 10 wt % of P_2_O_5_ in the points where SHERLOC detected 330- to 350-nm luminescence (Fig. 6). However, Thornton Gap and Berry Hollow did not yield enough data points for a statistical test.

**Table 1.** Summary of correlation statistics between PIXL elemental maps and survey scan SHERLOC 330- to 350-nm luminescence intensity maps. Rock targets from Fig. 5 are presented in rows. Columns show statistical metrics of Pearson’s correlation coefficient, *r*, and related *P* value as well as the structural similarity index (*SSIM*). Correlation statistics results for 330- to 350-nm luminescence and P_2_O_5_ are compared with a summary of the results for all other elements.

Target	*r* (lum.-P_2_O_5_)	*P* value (lum.-P_2_O_5_)	*r* (lum.-element)	*SSIM* (lum.-P_2_O_5_)	*SSIM* (lum.-element)
Bellegarde	0.6	10^−94^	<0–0.2	0.5	<0–0.2
Alfalfa	0.4	10^−44^	<0–0.1	0.4	<0–0.1
Montpezat	0.7	10^−58^	<0–0.2	0.6	0–0.2
Dourbes Scan 1	0.8	10^−105^	<0–0.4	0.8	<0–0.5
Dourbes Scan 2	0.8	10^−132^	<0–0.6	0.7	<0–0.4
Quartier	0.6	10^−94^	<0–0.2	0.7	<0–0.4
All	0.7	<10^−308^	<0–0.3	N/A	N/A

### Correlations of 330- to 350-nm luminescence and phosphate Raman detections

**Fig. 7.**
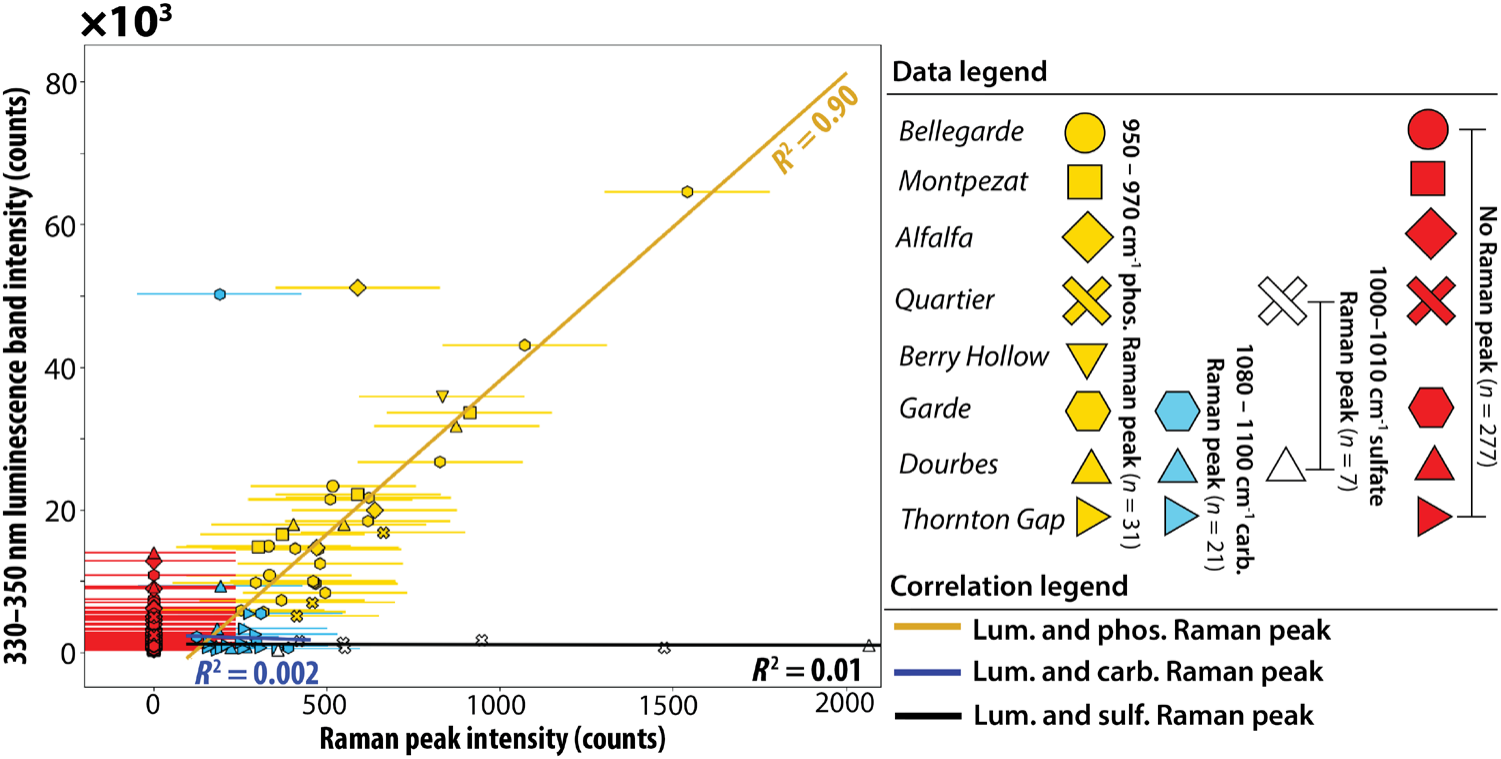
Correlation of phosphate, carbonate, and sulfate Raman peak intensities with the intensity of 330- to 350-nm luminescence across the different rock targets. Phosphate minerals (950 to 970 cm^−1^ Raman peaks; yellow) correlate with 330- to 350-nm luminescence, *R*^2^ = 0.90. Carbonate peaks (1080 to 1100 cm^−1^ Raman peaks; blue) and sulfate peaks (1010 to 1100 cm^−1^ Raman peaks; white) do not correlate with 330- to 350-nm luminescence (*R*^2^ ≤ 0.01). All carbonate and sulfate Raman peaks occur in points where the counts of the 330- to 350-nm luminescence are lower than 14,000 counts, except for one point in Garde. Error bars are derived from the range of variation in pixel counts from empty scans in which no material is measured (luminescence error bars are smaller than symbols).

To further test the predictions outlined in the “Summary of predictions” section (Figs. 2 and 3), we calculated the correlations between luminescence and Raman detections of phosphate and other minerals in high-dynamic range (HDR) and detail scans (250 to 500 pulses per point). As discussed above, SHERLOC should be able to detect the luminescence from Ce^3+^ phosphate grains that are smaller than the sizes of SHERLOC or PIXL spots (Fig. 4) even if it cannot detect a Raman signal (Figs. 2 and 4). To exclude false detections due to instrument noise, a signal-to-noise ratio (SNR) > 3 was used as a threshold for detection of Raman mineral peaks. The noise was estimated as the average pixel-to-pixel count range, 240 counts, in the Raman shift region between 800 and 1100 cm^−1^ when SHERLOC measures an empty target (i.e., with only air in the detection volume) (error bars in Fig. 7). Independently, we observe that Ce^3+^ in phosphate can be detected as 330- to 350-nm luminescence without detection of a phosphate Raman peak when the Raman signal is <250 counts (Fig. 7). Owing to this lower spatial resolution, HDR and detail scans do not allow for statistically robust correlations of SHERLOC Raman and PIXL maps. However, statistically significant correlations of luminescence with P_2_O_5_ from the survey scans without Raman information (shown in Fig. 5) should hold for the lower resolution scans from the very same areas of the same spectral signals.

In the six survey scans, 7776 points all had high degrees of correlation with phosphorus (Fig. 5). These scans do not provide any Raman information due to low laser integration time. There were 336 points in all HDR and detail scans (2400 scan points) of the same areas in all targets that exhibited 330- to 350-nm luminescence. Of these, only 31 points contained detectable phosphate Raman peaks, 28 other points contained sulfate or carbonate peaks, and 277 points contained no mineral Raman peaks (Fig. 7) but do contain PIXL detections of phosphorus (Fig. 6 and figs. S5 and S7). The trends captured in this diagram (Fig. 7) match the predictions for Ce^3+^ luminescence from phosphate grains with variable grain sizes (Figs. 2 and 3) and from ACRONM laboratory measurements (Fig. 4). PIXL P_2_O_5_ maps demonstrated the presence of 3 to 10 wt % P_2_O_5_ without detected phosphate Raman peaks in all HDR and detail scans (Fig. 6 and figs. S5 and S7). This suggests that SHERLOC does not detect Raman peaks in all areas where phosphate is present (Figs. 2 and 3). The 31 points that did contain phosphate Raman peaks had the most intense 330- to 350-nm luminescence (>10,000 counts) out of all points (Fig. 7) and the intensities of the Raman peaks and luminescence were linearly correlated (Fig. 7). This linear correlation and high-intensity luminescence were not observed in 28 points where sulfate or carbonate Raman peaks were detected. Points with sulfate, carbonate, or no peaks exhibited lower-intensity (<10,000 counts) 330- to 350-nm luminescence with only a few instances where the intensity of the luminescence matched that of 330- to 350-nm luminescence co-occurring with detected phosphate Raman peaks. This data pattern closely matches the prediction for Ce-bearing phosphate as the source of SHERLOC luminescence (Figs. 2 and 3). Only two points in which 330- to 350-nm luminescence co-occurred with phosphate and with carbonate were notably offset from the correlation trends (Fig. 7). The point that co-occurred with a phosphate Raman peak is likely still from Ce in phosphate grain that was shifted from the general phosphate correlation line for unknown reasons. The luminescence associated with the carbonate Raman detection did not exhibit an unusual spectral shape but constitutes an ambiguous signal.

The analyses presented so far show strong correlations among SHERLOC 330- to 350-nm luminescence, phosphate Raman peaks, and PIXL P_2_O_5_ concentrations (Fig. 7). Therefore, to search for robust signals of putative naphthalene-like or macromolecular organic compounds contributing to 330- to 350-nm luminescence, we examined locations where PIXL did not observe P_2_O_5_ and SHERLOC did not observe phosphate Raman peaks (fig. S12). Such luminescence signals (fig. S12) would be the strongest candidates for the potential presence of two-ring aromatic compounds in the rocks from Jezero crater. All the 121 points in Bellegarde, Berry Hollow, and Garde that showed 330- to 350-nm luminescence were associated with a phosphate Raman peak or P_2_O_5_ PIXL detection. In the remaining Thornton Gap, Dourbes, Alfalfa, and Montpezat scans, 4 of 15, 6 of 48, 11 of 64, and 14 of 27 luminescing points covered by PIXL maps, respectively, did not occur with P_2_O_5_ hotspots and lacked associated phosphate Raman peaks (fig. S12).

We examined these 35 points in Thornton Gap, Dourbes, Alfalfa and Montpezat targets for signals that are diagnostic of macromolecular organic compounds (fig. S12). First, we searched for low-intensity luminescence at 330 to 350 nm without the double Gaussian mixture shape predicted for Ce^3+^ (e.g., signals with a smooth positive slope) (e.g., Fig. 1A, dark green spectrum). We were unable to identify such features in any of these scan points (fig. S12). The width, band positions, and shape components could not be differentiated from 330- to 350-nm luminescence associated with phosphate Raman peaks and P_2_O_5_ (fig. S12C). All these points also exhibited comparatively low-intensity luminescence (<4000 counts) and lacked Raman G-bands. Given that we could not distinguish these signals from phosphate-associated luminescence, we suggest that they may be (i) luminescence signals from Ce-bearing phosphate that is not detectable by PIXL and SHERLOC due to the small grain size or the correlation is not apparent due to inherent uncertainties in the coregistration methodology (Materials and Methods), (ii) luminescence signals from Ce in other minerals, or, in fact, (iii) they are organic luminescence. Because the 330- to 350-nm luminescence due to 5d-4f transitions in Ce^3+^ is documented to occur in carbonates, sulfates, zircons, and silicate minerals [e.g., (*39*) and references herein], our favored hypothesis is (ii).

### Correlation between 270- to 290-nm luminescence, silica, and other phases

To test the predictions outlined in the “Summary of predictions” section, we investigated correlations between silica/silicates and 270- to 290-nm luminescence. Testing for possible correlations of 270- to 290-nm luminescence with mineral phases is challenging because of the smaller dataset (table S1). Only two targets, the Bellegarde and Alfalfa targets, contained enough analytical spots with 270- to 290-nm luminescence for quantitative analysis. Luminescence with the maximum at 270 to 290 nm occurs both with and without 330- to 350-nm luminescence throughout the crater floor targets (Fig. 1). In the Garde target, 270- to 290-nm luminescence always co-occurs with 330- to 350-nm luminescence. The intensities of 270- to 290-nm and 330- to 350-nm luminescence do not correlate with each other (fig. S13). Therefore, the two types of luminescence are best explained by at least two different emitters.

**Fig. 8.**
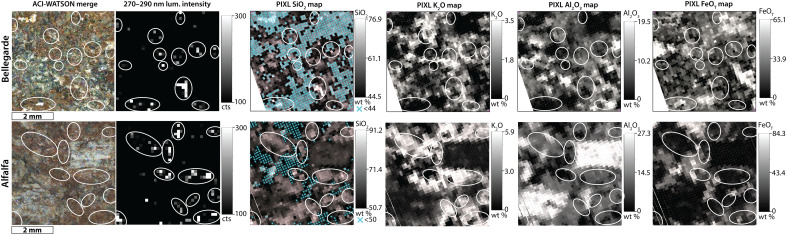
Comparison between 270- to 290-nm luminescence and PIXL elemental maps in the Bellegarde and Alfalfa targets. Left panels show colorized ACI-WATSON image merges. Grayscale heatmaps show 270- to 290-nm luminescence intensity in counts, and white ellipses in all maps outline areas with major 270- to 290-nm luminescence. The last four columns show PIXL SiO_2_ wt %, K_2_O wt %, Al_2_O_3_ wt %, and FeO_T_ wt % maps. Blue crosses in SiO_2_ wt % PIXL maps signify that SiO_2_ contents are below the given threshold values of 44 wt % and 50 wt %. Nearly all pixels that exhibit 270- to 290-nm luminescence occur within Si-rich phases (SiO_2_ > 75 wt %) and K- and Si-rich phases (SiO_2_ > 50 wt %, K_2_O > 3 wt %). Ovals highlight the location of the 270- to 290-nm luminescence in all images, luminescence maps, and element maps.

Targets from the Séítah fm (Garde and Dourbes) and the sedimentary fan front (Thornton Gap and Berry Hollow) contained type 2 luminescence centered only at 285 to 295 nm, whereas luminescence in the Máaz fm targets spanned the full range of 270 to 295 nm. This luminescence was detected primarily in the Máaz fm targets that were characterized by higher SiO_2_, P_2_O_5_, and K_2_O contents compared to the Séítah fm and sedimentary fan front (table S1). In Bellegarde, one high-intensity cluster of 270- to 290-nm luminescence correlated spatially with a high-Si phase (>75 wt % SiO_2_) (Fig. 8). The remaining lower-intensity luminescence was variably spread across the fine-grained groundmass composed of K- and Si-rich phases (SiO_2_ > 50 wt %, K_2_O > 2 wt %) (Fig. 8). Analyses of the Alfalfa target localized the 270- to 290-nm luminescence to the reddish-brown fine-grained K- and Si-rich phases (SiO_2_ > 50 wt %, K_2_O > 3 wt %) and high-Si phase (~80 to 90 wt % SiO_2_) (Fig. 8). Statistical analyses indicate a slight correlation between 270- and 290-nm luminescence and SiO_2_ in both targets (*r* = 0.2, *P* value = 10^−9^ to 10^−10^, *SSIM* = 0.2 to 0.3) and a correlation between the 270- to 290-nm luminescence and K_2_O in the Alfalfa target (*r* = 0.2, *P* value = 10^−9^, *SSIM* = 0.2) (table S3). However, the weak statistical correlation reflects that most SiO_2_ and K_2_O material does not contain 270- to 290-nm luminescence as best evaluated through inspection of Fig. 8. This would be expected for either defect or organic compounds.

The same patterns were observed in HDR scans of Alfalfa and Bellegarde, with the additional 270- to 290-nm luminescence observed inside the larger feldspar grains of Alfalfa (fig. S6). These signals and correlations suggest that the 270- to 290-nm luminescence cannot be easily assigned to one-ring aromatics without improving the current methodology for differentiating defect luminescence and organic luminescence.

### Summary of comparison between observations and predictions

The following points are summarized in Table 2. Type 1 and type 2 signals refer to descriptions given in the “Predictions of Organic and Inorganic Hypotheses” section. (i) No Raman G-bands, except one reported detection, are observed in the datasets. This observation contradicts the expectation for an organic origin of luminescence. (ii) We observed a spatial correlation between phosphate detected by PIXL and type 1 luminescence. This observation contradicts the expectation for an organic origin of luminescence. (iii) We observed a spatial correlation between phosphate detected by SHERLOC Raman and type 1 luminescence. This observation contradicts the expectation for an organic origin of luminescence. (iv) We observed a spatial correlation between silica and K-bearing silicates and SHERLOC type 2 luminescence. Therefore, an inorganic origin of type 2 luminescence cannot be excluded based on correlations. (v) We did not observe any differentiable spectral shape components from Gaussian doublet and single Gaussian shapes for type 1 and type 2 luminescence, respectively. Therefore, an inorganic origin of the luminescence observation cannot be excluded based on correlations.

**Table 2.** Table of predictions observed or not observed on Mars. Predictions for the different proposed sources of 330- to 350-nm luminescence based on laboratory analog instruments. Rows represent the four most important hypotheses for the sources of 330- to 350-nm luminescence considered in this study. Columns show expected wavelength position of the maximum intensity (λ_max_), expected band shapes from measurement of standards so far, expected correlation trend with P_2_O_5_, expected correlation trend with phosphate Raman peaks, and expected correlation trend with Raman G-band for each of the hypothesized sources. Observations made on Mars with the SHERLOC and PIXL instruments are shown in bold font, while observations not made on Mars are shown in normal font. Observations that are partially but not fully fulfilled are shown in bold with a corresponding footnote. Observations that require recommended additional work for a comprehensive evaluation are shown in italics.

Potential sources (type 1 lum.)	λ_max_ (nm)	Raman G-band	Spatial correlation with P_2_O_5_ abundance	Spatial correlation with phosphate Raman peak	Band shape components at SHERLOC resolution
Organic	**~330–350**	*Present for ~>0.1 wt % carbon*	High-intensity luminescence without P_2_O_5_ correlation	High-intensity luminescence without phosphate detection correlation	Any lum. shape that cannot be explained by Gaussian doublet (including but not limited to vibronic bands or positive slope)
Ce^3+^ in phosphate	**~330–350**	**Should not be present**	**Statistically significant correlation between luminescence and P**_**2**_ **O**_**5**_	**Statistically significant correlation between phosphate Raman peaks and luminescence**	**Asymmetric Gaussian shape indicate ~340**-**nm and ~360**-**nm Gaussian doublets**
Potential sources (type 2 lum.)	λ_max_ (nm)	Raman G-band	Spatial correlation with silica and aluminosilicates	Spatial correlation with mineral Raman peaks	Band shape components at SHERLOC resolution
Organic	~280–300*	*Present for ~>0.1 wt % carbon*	Can be present with silica/silicates but no correlation would strongly indicate organics*	Luminescence correlated to other minerals than silica/silicates†	Any lum. shape that cannot be explained by Gaussian singlet (including but not limited to vibronic bands or positive slope)
Silica/silicate defect	**270–290**	**Should not be present**	**Exclusive presence of luminescence with silica/aluminosilicate**	**No observation of correlated Raman peaks because silica and silicate primary modes are outside the Raman wavelength**	**Gaussian singlet**

*These criteria are partially fulfilled.

†One exception found in the Garde rock (fig. S7); however, no PIXL data are available for investigation of co-occurring silica/silicate phases.

## DISCUSSION

Previous studies (*8*, *9*) expressed the need to better understand the origins of UV luminescence detected by SHERLOC in natural samples from Jezero crater, especially as motivated by laboratory studies (*8*, *49*, *50*). This study responds to this need by proposing and developing criteria (Figs. 2 and 3 and Table 2) that can improve the confidence of potential organic detections. On the basis of these criteria, areas that exhibit low-intensity luminescence at 330 to 350 nm or 270 to 290 nm and lack detectable phosphorus-containing or silica-, aluminosilicate-, or feldspar-containing materials are the best candidates for detecting small amounts of organic compounds. Conversely, the strong correlation of 330- to 350-nm luminescence with the detections of phosphorus by PIXL indicates that Ce^3+^ in phosphate may be the source of most, if not all, of the 330- to 350-nm luminescence detected thus far in Jezero crater (Table 2). This correlation and the SHERLOC-detectable UV luminescence of Ce^3+^ in other minerals such as sulfate (*14*, *21*) present in Jezero crater rocks mean that the presence of Ce^3+^ in minerals should be the null hypothesis related to the SHERLOC detections of UV luminescence. UV luminescence with maxima between 270 and 290 nm is less common and its origin is harder to constrain, but its association with materials that contain silica, K-silicate phase, and feldspars presents an additional inorganic hypothesis to test before assigning an organic origin to these luminescence signals (Table 2). The rover instruments cannot independently confirm the presence of such defects or one-ring aromatics, so these confirmations require return of the samples. Future analog studies that measure the UV luminescence of defects in naturally occurring plagioclase, Fe-poor clay minerals or amorphous silicates, and silica such as opal can elucidate the expected prevalence of luminescent defects in natural martian materials and conditions.

In addition to measuring the luminescence, SHERLOC can search for organic Raman features, such as the macromolecular carbon G-band (*4*). This Raman feature, if present in multiple analysis points and at a high SNR, would provide evidence for organics (Fig. 2 and Table 2) (*4*, *7*, *10*, *41*, *47*, *48*). To date, only 1 of 1800 scan points on the crater floor has a reported G-band detection (*9*, *13*) and Raman G-bands have not been reported in the 600 points from any sedimentary fan front materials analyzed in this study. This points to the low organic content of the rocks from Jezero crater floor and sedimentary fan front analyzed thus far. In what follows, we assess the current knowledge of SHERLOC G-band detectability as compared to Mars organic compound concentrations.

Previous studies (*8*, *9*) used the SHERLOC optical model from (*4*) to estimate that Jezero crater floor rocks contain 0.1 to 20 ppm organics under the assumption that the detected luminescence emanated from one- and two-ring aromatic compounds, but it did not produce a G-band. This estimate is lower than the typical organic content of fine-grained, aqueously deposited sedimentary rocks from Earth that preserve UV Raman-detectable organic matter and morphological biosignatures [e.g., (*10*, *41*, *47*)]. For example, organic-rich lamina and microfossils within fluviolacustrine Archean silty and sandy shales contain 0.1 to 0.6 wt % carbonaceous materials (*65*) and the silica-containing Archean Buck Reef Chert has a 0.1 to 0.2 wt % organic content (*66*). The Curiosity rover does not contain a Raman spectrometer, but its sensitive gas chromatography and mass spectrometry showed that aqueously deposited siliciclastic mudstones and sandstones in Gale crater, Mars, contain organic matter mainly in macromolecular form at concentrations of 70 parts per billion to potentially hundreds of ppm (*33*, *34*, *67*, *68*). The content of reduced carbon in igneous rocks from Mars is more difficult to estimate because unambiguously igneous rocks are not reported in Gale crater and martian meteorites are contaminated by terrestrial organic compounds (*12*).

The reported limit for detecting Raman features of aliphatic, aromatic, and condensed carbon class of organics by SHERLOC is reported to be 0.1 wt % within a 100-μm area in (*4*). However, detection depends on many instrument- and material-dependent factors that we recommend be tested. This could be tested by using an analog model of SHERLOC to measure standards with well-constrained organic contents in a series of experiments that systematically alter measurement conditions to fit Mars-specific scenarios. In practice, SHERLOC can detect Raman bands attributed to graphitized organics (G-bands) and the accompanying luminescence signatures in one of its calibration targets, the SaU 008 martian meteorite, and the Allende carbonaceous chondrite (*4*, *48*). A laboratory analog instrument to SHERLOC (different from ACRONM) can also measure a well-defined Raman G-band and ~305-nm luminescence in exceptionally preserved microbial textures in Buck Reef Chert containing ~0.1 wt % bulk carbon (*47*), suggesting that Jezero rocks contain less organic matter than the Buck Reef Chert. Thus, if Jezero rocks contain organic compounds at similar abundances and in similar macromolecular forms as those in terrestrial rocks or SHERLOC’s on-board meteorite standards, SHERLOC would be able to detect them. However, if these compounds typically occur at bulk concentrations below ~100 ppm, as suggested by the Curiosity measurements, and are not spatially concentrated in areas comparable to the SHERLOC spot sizes, they could evade detection.

Given the expectation that most organic matter in martian rocks should be in macromolecular form (*33*, *34*), it is unlikely that SHERLOC would detect pure one- or two-ring aromatics as the primary sources of luminescence and Raman signatures in Jezero rocks. However, if present, materials that occur at such abundances would be readily measurable in terrestrial laboratories and would be of enormous astrobiological value upon sample return. Rocks from the early Earth contain thermally mature, very recalcitrant organic matter [e.g., (*69*)] that has lost most soluble compounds that carry structural, compositional, and isotopic signals of formative and early diagenetic processes. In contrast, the detections of aqueously deposited and hydrated minerals by Perseverance [e.g., (*8*, *16*–*22*)] indicate that any organic matter associated within these phases would have much lower thermal maturity and therefore a higher potential to retain some soluble components, perhaps in a manner similar to meteorites [e.g., (*12*, *31*, *32*)]. Even if SHERLOC luminescence signals are predominantly inorganic, this does not preclude or affect opportunities for finding diverse organic compounds in the collected samples upon return to Earth, where they can be studied with highly sensitive laboratory techniques [e.g., (*1*–*3*, *16*, *21*)].

This study has established the following points:

1) Luminescence at 330 to 350 nm correlates with phosphate abundances in Jezero rocks, indicating that cerium-bearing phosphate grains are the dominant source of this luminescence.

2) The association of 270- to 290-nm luminescence with high SiO_2_ and K_2_O and a single Gaussian spectral profile suggests that silica and silicate defects are potential sources of this luminescence.

3) SHERLOC does not measure the 400- to 500-nm wavelength range, which is the luminescence maxima of the most common extraterrestrial organic materials, but also includes a lot more metal interference than just Ce^3+^.

4) The near total absence of Raman G-band detections by SHERLOC indicates that rocks analyzed in the Jezero crater floor and lower sedimentary fan contain <0.1 wt % organic carbon. Organic compounds at lower concentrations on Mars could still be present in these samples.

5) Only analyses of returned samples can resolve the origin of detected luminescence and characterize organic compounds that may be present in the Jezero rocks analyzed in this study.

## MATERIALS AND METHODS

### Laboratory analyses of Raman and luminescence signals in NWA 10922

The Raman and luminescence spectra of the martian meteorite NWA 10922 were collected using the ACRONM instrument, which is a SHERLOC analog instrument located at the NASA Johnson Space Center. ACRONM uses a PhotonSystems NeCu70-248 hollow cathode laser to produce a 248.5794-nm pulsed incident laser beam operated at a repetition rate of 20 Hz, a pulse width of 40 μm, and laser energy of ~4.7 μJ pulse^−1^. The incident light is focused on a ~75-μm spot on the sample’s surface using a 5× objective. Raman scattering and luminescence are collected and collimated through the 5× objective using a 180° backscattering geometry and sent to a Horiba Scientific iHR 320 spectrometer to disperse the light before being detected using a back-thinned Scientific Synapse Plus CCD (SYN-PLUS-2048X512-BU) thermoelectrically cooled to −75°C. For the collection of Raman spectra, a 2400 g/mm grating was used to provide a resolution of ~2.15 cm^−1^ per pixel and a spectral range of ~0 to 4500 cm^−1^. For the collection of luminescence spectra, a 300 g/mm grating was used to provide a resolution of ~0.18 nm/pixel and a spectral range of ~240 to 520 nm.

Raman and luminescence spectra were collected at phosphate locations of NWA 10922 identified by XRF. For each location, six Raman spectra were collected at slightly different beam positions within the phosphate-bearing area. Each Raman spectrum was collected using six 10-s accumulations, and similar Raman spectra collected within a phosphate location were averaged to improve SNR. For each phosphate location, 10 luminescence spectra were collected. Each luminescence spectrum was collected using ten 0.1-s accumulations. When the luminescence signal was strong enough to saturate the detector, 0.01-s accumulation times were used. Similar luminescence spectra for a given phosphate location were averaged to improve SNR and average out intensity variations in the luminescence spectra. Raman spectra were calibrated using cyclohexane and the atmospheric N_2_ stretching band. Luminescence spectra were calibrated using a Mercury-Argon emission lamp.

Elemental distributions of a sawed and polished surface of NWA 10922 were mapped using a Bruker M4 Tornado Plus benchtop micro–x-ray fluorescence (μXRF) instrument (Bruker Nano GmbH, Berlin, Germany). The μXRF instrument is equipped with a 30-W rhodium anode x-ray tube operated at 50 kV and 600 μA and two 60-mm^2^ silicon drift detectors (SSDs) under 2-mbar vacuum conditions. The x-ray beam is focused by a polycapillary lens into an approximately 20-μm spot size with no beam filtering. Elemental distribution maps were acquired with a 17-μm pixel size and a 15-ms/pixel integration time. The surface of the slab was approximately 36 mm × 36 mm (a total of 4.4 million pixels) and took around 10 hours to complete the x-ray map. Each pixel has an associated XRF spectrum, and the spatial distribution of a single element, if detectable, can be isolated and mapped as a function of its characteristic kα peak.

### SHERLOC acquisition of flight data

SHERLOC is a deep UV spectrometer that can detect both organic compounds and minerals using Raman spectroscopy (*4*). The instrument can also detect luminescence signals with organic and inorganic sources (*4*, *7*–*9*). SHERLOC uses a 110-μm-diameter (equivalent to spot size) UV 248.6-nm pulsed laser to scan the surface in a grid (*4*). The UV laser excitation source enables the separation and analyses of the luminescence and Raman scattering wavelength windows (*4*). This separation aids in the characterization of the Raman region because Raman scattering produces a much weaker signal than luminescence. Fe-containing materials can absorb the UV radiation, thereby leading to attenuation of the Raman and luminescence signals as well as false-negative detections (*4*, *6*). The SHERLOC instrument characterizes the Raman region from ~800 to 4000 cm^−1^ with a pixel resolution of ~10 cm^−1^ and an average Raman shift position uncertainty of ±5 cm^−1^ depending on how well peaks are resolved and modeled (*4*). The instrument characterizes the luminescence region from 250 to 355 nm.

SHERLOC uses different scan types for targeting luminescence and Raman due to their outlined sensitivity differences. SHERLOC survey scans target luminescence only with 15 laser pulses per point for 36 × 36 points over a 5 mm × 5 mm area. To enable the acquisition of both Raman scattering and luminescence signals, SHERLOC uses HDR and detail scans with fewer raster points compared to SHERLOC survey scans but with 250 to 500 laser pulses per point. HDR scans contain 10 × 10 scan points over 7 mm × 7 mm while detail scans contain 10 × 10 scan points over 1 mm × 1 mm.

All targets were abraded by the Perseverance rover’s abrasion tool before characterization by the SHERLOC instrument and the abrasion patches were cleaned with the gas dust removal tool to provide a clean, flat rock surface (*1*). The SHERLOC instrument is mounted on the robotic arm of the Perseverance rover together with the Autofocus Context Imager (ACI) (10.1 μm per pixel, grayscale) and the WATSON camera (16 to 150 μm per pixel, color) (*4*). The cameras are used to take high-resolution images of the abraded rock before each SHERLOC scan. Then, SHERLOC scans the abraded rock using three types of grid scan templates: (i) survey, (ii) HDR, and (iii) detail maps. Survey maps use 15 pulses per point (ppp) in a 36 × 36 grid over a 5 mm × 5 mm area. HDR maps use 500 ppp arranged in a 10 × 10 grid over a 7 mm × 7 mm area for most targets. The Bellegarde rock was analyzed with 250 ppp. Detail scans are measured with 500 ppp in a 10 × 10 grid over a 1 mm × 1 mm area. Survey scans produce the most data and can easily resolve strong luminescence features but typically yield little to no Raman signal. HDR (500 ppp) and detail scans can analyze for the presence of both luminescence and Raman features. The first two rocks on Mars, Bellegarde and Guillaumes, were measured with 250 ppp, after which other rocks were measured with 500 to 900 ppp to increase the Raman signal. The 250 ppp in the Bellegarde HDR scan likely precluded analyses of Raman features, because Raman signal is dependent on integration time measured as laser pulses per point. For each scan point, an active and a dark spectrum are acquired, and the dark spectrum is subtracted from the active. This subtraction product results in the spectrum that is subsequently analyzed. The spectra were calibrated according to methods in (*48*), resulting in an estimated uncertainty of ±2 cm^−1^ from calibration alone.

### Analyses of SHERLOC spectra

This study used four different modes of spectral analysis: (i) visual identification of luminescence and Raman features in all scan types; (ii) luminescence intensity heatmaps of survey and detail scans; (iii) modeling to extract maximum intensity (*I*_max_), wavelength of *I*_max_ (λ_Imax_), and full width at half maximum (FWHM) of all 270- to 290-nm and 330- to 350-nm luminescence features in HDR and detail scans; and (iv) Raman peak identification and fitting, and *I*_max_ extraction of known mineral Raman peaks.

#### Visual identification

All 270- to 290-nm and 330- to 350-nm luminescence features that were distinguishable from the baseline in HDR and detail scans were identified as a qualitative check for the luminescence heatmaps and Gaussian mixture modeling. The locations of these features in grid scans were visualized by different colored symbols (Fig. 6 and figs. S5 to S8).

#### Luminescence intensity maps

SHERLOC survey and detail scans yielded grayscale luminescence intensity heatmaps with 144- and 100-μm distances between scan points, respectively. These distances match the 110-μm diameter of laser spots. The SHERLOC survey scan was thus reprocessed to a 0 to 255 normalized 36 × 36 pixel grayscale heatmap of the rolling mean values in a particular wavelength range. We used the wavelength window of 344 to 346 nm to track 330- to 350-nm luminescence intensity and the wavelength window of 280 to 290 nm to track 270- to 290-nm luminescence intensity. An additional processing step used a manually determined threshold to check for the presence of cosmic rays. If the processing step identified high-intensity points due to cosmic rays, the intensity was set to 0 within the image. The same procedure re-processed detail scans to 10 × 10 pixel grayscale heatmaps. For detail scans, every single spectrum was investigated visually to remove false positives from cosmic rays. The heatmaps of survey and detail scans were subsequently analyzed together with PIXL grayscale elemental oxide maps as detailed. Pixelated maps generated in this manner are not an appropriate visualization method for HDR scans because the distance between points for HDR scans is 700 μm. Therefore, statistical comparisons between HDR and elemental maps are not performed; however, overlay comparisons are described.

#### Luminescence modeling

We performed luminescence modeling on all 270- to 290-nm luminescence and 330- to 350-nm luminescence from all HDR and detail scans. HDR and detail scans are taken over the same area and materials as survey scans. Therefore, these are taken to represent higher intensity and better resolved variations of the same luminescence signals. Before Gaussian mixture modeling, we deployed a simple algorithm for *I*_max_ and λ_Imax_ extraction. The spectra were smoothed using either rolling means or the scipy Savitzky-Golay filter (*70*), depending on the noise profile of the spectrum. *I*_max_ and λ_Imax_ were extracted between 270 to 295 nm and 325 to 350 nm. The similarity between the raw and smoothed spectra as well as the extracted *I*_max_ and λ_Imax_ were evaluated visually for all spectra. Cosmic rays that affected the extracted numbers were removed. The smoothing algorithms did not perform well when the luminescence features had low intensity (approximately <400 counts) due to the noise, so other smoothing algorithms were applied without much success. *I*_max_ and λ_Imax_ were extracted manually from such spectra. Count cutoffs based on the approximate background counts of each individual rock scan were used to filter out particularly low-intensity luminescence features that are only barely discernible from background. Luminescence with a maximum at 330 to 350 nm in Bellegarde and Montpezat was not evaluated when the features had fewer than 400 counts; in Garde, this cutoff was <500 counts. In Dourbes, all 330- to 350-nm bands had more than 450 counts and were clearly discernible from the background. For 270- to 290-nm luminescence, spectra with less than 300 and 400 counts, respectively, were not evaluated for Bellegarde and Alfalfa/Dourbes, respectively. These cutoffs were 200 and 500 counts for Montpezat and Garde.

Shape differences in the 330- to 350-nm luminescence bands within HDR and detail scans were analyzed by Gaussian mixture modeling. We used the lmfit module for single Gaussian model fits and double Gaussian mixture fits (*71*). All 330- to 350-nm luminescence bands could be fully modeled by either a single Gaussian centered between 340 and 350 nm or a Gaussian mixture with two Gaussians centered at ~330 nm and ~340 to 360 nm. The position of the second Gaussian has considerable uncertainty because it is only partially resolved in the SHERLOC wavelength range window. Residuals were evaluated for best fit; FWHM, *I*_max_, and λ_Imax_ values were extracted for all fits. *I*_max_ and λ_Imax_ were then compared to those modeled through smoothing or manual extraction. The use of Gaussian mixture models was tested on luminescence data acquired on NWA 10922 in the laboratory: The residuals were low and the approach performed well. However, fitting 330- to 350-nm luminescence bands with Gaussian mixture models is a nonunique problem that does not yield unique solutions. This is especially true for flight data because the SHERLOC wavelength range captures only a portion of the data that are required for full Gaussian mixture modeling (Fig. 1). Hence, this type of modeling can only be used as a method for extracting some common shape components, such as *I*_max_, λ_Imax_, and FWHM*.*

#### Raman peak fitting

All Raman peaks identified in map representations of Bellegarde and Garde targets were previously processed and analyzed by Scheller *et al.* and Corpolongo *et al.* (*8*, *13*). Alfalfa, Montpezat, and Dourbes targets were analyzed by Corpolongo *et al.* (*13*) only. This study includes detections of Raman peaks in Thornton Gap and Berry Hollow. The standard methods for the identification of peaks in the Raman spectra acquired by SHERLOC in all these studies include (i) baseline fitting and subtraction, (ii) Gaussian fitting to the Raman region of interest, (iii) a measure of noise, and (iv) calculation of SNR and model performance metrics.

1) Background subtraction: The background was fitted using the pybaselines Small-Window Moving Average (SWIMA) algorithm (*72*). A collection of pybaseline algorithms were tested on SHERLOC data, including those based on polynomial fits and Whittaker methods. Smoothing-based approaches, like SWIMA, were found to perform best on the SHERLOC background. Window parameters are adjusted to provide the best baseline fit.

2) Gaussian fitting and model performance: Gaussian fitting of Raman peak regions is performed using the curve fit algorithm from Scipy (*70*). Peak position, intensity (*I*_max_), FWHM, and model fit squared residual (*R*^2^) are extracted. *R*^2^ > 0.7 and FWHM >3 pixels is used to qualify a peak fit and to distinguish Raman peaks from cosmic rays.

3) Noise measure: The standard definition of noise on the SHERLOC spectrometer is the SD of the background in a defined silent region from 2000 to 2100 cm^−1^ (σ_silent_) within which Raman features are unlikely to appear. In addition, we calculate the SD of the Raman region of the signal from the stowed arm scan (σ_stowed_) for comparison. The stowed arm scan approximates an on-flight blank measurement in which the instrument measures an empty space with nothing in focus in order to characterize noise. Both are compared to peak intensity to evaluate performance. However, σ_silent_ is used for standard SNR calculations.

4) SNR: A measure of SNR can be evaluated based on the definition in (*73*).

$$\mathrm{SNR}=\frac{S}{\sigma_{y}}$$

$$\sigma_{y}={\left({\sigma_{S}}^{2}+{\sigma_{B}}^{2}+{\sigma_{d}}^{2}+{\sigma_{F}}^{2}+{\sigma_{r}}^{2}\right)}^{1/2}$$

Here, *S* is the signal intensity, σ_s_ is the SD of signal from the band of interest, σ_B_ is the SD of the background, σ_d_ is the SD of the dark signal, σ_F_ is the SD of the flicker noise, and σ_r_ is the SD of readout noise. Here, we adapt a simplified expression that is a practical encapsulation of background, dark signal, and flicker noise

$$\mathrm{SNR}=\frac{I_{\text{max}}}{\sigma_{\text{silent}}}$$

This practical implementation is made because we cannot afford repeated measurements of the same target and bracketing blank measurements during in-flight measurement. Therefore, we use a standard region with no Raman features as an approximation of our noise sources. This is further supplemented with an identical comparison to σ_stowed_ in bracketing blank measurements, which are occasionally made as well. This method approach led to 1075 to 1100 cm^−1^ detections interpreted as carbonate, 1010 to 1020 cm^−1^ detections interpreted as sulfate, and one 950 cm^−1^ peak for Thornton Gap and Berry Hollow scans (Fig. 6). The only Raman feature that is not reported in this study is a broad ~1060 cm^−1^ feature that was identified visually in (*8*, *13*).

The Raman peaks of particular interest to this study are located at 950 to 970 cm^−1^. Both phosphate and a variety of perchlorates and chlorates can have overlapping primary mode positions in this range and SHERLOC does not detect any secondary modes for these minerals in most cases. Hence, the 950 to 970 cm^−1^ Raman peaks are described as either potential perchlorate or potential phosphate in (*8*, *13*). Laboratory measurements using the ACRONM instrument in (*8*) and this study show that apatite peak positions common to martian materials range from 950 to 970 cm^−1^ at SHERLOC-like resolution (fig. S4). Using an uncertainty estimate for any Raman peak position of ±5 cm^−1^, the allowable range for chloro- and fluorapatite Raman peak positions is therefore 945 to 975 cm^−1^ for the SHERLOC instrument.

For Fig. 7 specifically, the maximum intensity *I*_max_ of 950 to 970 cm^−1^ Raman peaks identified in HDR and detail scans were extracted directly from the raw spectrum after subtracting the background using methods described in the “1. Background subtraction” paragraph earlier. Gaussian fitting was not used for intensity extraction, as we were interested in the raw signal as opposed to the modeled peak intensity. We note that all scan points outside the Quartier target featuring 950 to 970 cm^−1^ Raman peaks also featured the strongest 330- to 350-nm luminescence bands across all scan points (Fig. 7). Only in the Quartier target did two 950 cm^−1^ Raman peaks occur without 330- to 350-nm luminescence, which are not plotted in Fig. 7. We note that three 950 cm^−1^ Raman peaks in the same Quartier scans did coincide with 330- to 350-nm luminescence and are plotted in Fig. 7. All of these occurred inside a sulfate- and perchlorate-dominated material in Quartier pointing to a complicated material mixture (*13*, *14*).

### PIXL analysis and spectra processing

The PIXL instrument is an x-ray fluorescence spectrometer mounted on the arm of the Perseverance Rover. PIXL produces high-resolution (~120 μm spot size) elemental abundance maps by rastering its x-ray beam across the rock surfaces of interest (*15*). This paper includes eight PIXL scans across eight different outcrops as detailed in the main text (further information and direct link to datasets are found in Supplementary Text). Before analysis, an abrasion is carried out in the same fashion as described for the SHERLOC instrument. PIXL maps were collected with areas ranging between 5 mm × 7 mm, 7 mm × 7 mm, and 4 mm × 12.5 mm with a 0.125-mm step size. The x-ray system dwelled for 10 s at each point during XRF mapping, acquiring an energy-dispersive x-ray fluorescence spectrum in each of PIXL’s two detectors (*15*).

PIXL elemental quantification maps were generated using the PIQUANT XRF quantification program (*74*, *75*) and visualized with the open-source visualization software, PIXLISE (*76*). To register, or map, each single point of XRF measurement into the context image from the Micro Context Camera (MCC), PIXLISE considers the optical and geometric calibration of PIXL, surface topography, and positional drift of the instrument taking place throughout PIXL’s scanning (*15*, *76*–*79*). PIQUANT calculates the elemental weight % for each PIXL histogram using a physics fundamental parameters method, which takes into consideration histogram peak area, bremsstrahlung background, PIXL instrument and sample geometry, and known x-ray emission peak locations and relative intensities (*74*, *75*). Elemental quantification maps from combined detectors were exported as color maps for figures and as grayscale maps for correlation statistics. We compared the default quantification PIXLISE maps to further corrected maps and found no statistically significant differences that would interfere with the results of this analysis.

### SHERLOC-PIXL coregistration and correlation statistics

All SHERLOC grids are accompanied by grayscale images from an ACI and all PIXL elemental maps are accompanied by either raw grayscale images or four-channel multispectral images (UV, blue, green, and near-infrared) using the MCC (*77*–*79*). This study enabled the comparisons of SHERLOC and PIXL datasets by coregistering the respective context images of ACI and MCC, and thereby providing a coregistered spectral dataset map from both instruments. Optical features in ACI and MCC images were matched using the standard ENVI software tool that allows manual identification of matching points between the two images. ACI were taken at 10 μm per pixel (*4*) and MCC at 52 μm per pixel resolution (*15*) over the same location. ACI views the surface from an approximate normal to the surface, whereas the MCC’s viewpoint is tilted 18° from the surface normal. ENVI software warps the MCC images and corresponding PIXL elemental maps to match the ACI and corresponding SHERLOC grids (Fig. 5). Following the selection of 20 to 30 matching points that have a root mean square (RMS) error < 5 pixels for the MCC image, a polynomial warp function was applied to the PIXL MCC and all PIXL elemental maps with nearest neighbor resampling provided by the ENVI software. Such a warping assumes a planar surface and does not consider any topographic variations. However, for this analysis, a planar approximation is assumed to be sufficient as the topographic variation from the roughest regions of the scanned abrasions is in the order of 100 to 200 μm.

Each of the coregistered scans presented will contain a variable placement uncertainty of the x-ray point relative to the optical context captured by the MCC and further relative to the luminescent point. It typically affects the stem from surface topography and the positional drift of the instrument relative to surface. The reported in-flight correlation between PIXL’s XRF and optical context from MCC is in the order of tens of micrometers (*78*) as positional drift is measured and compensated for. Cross-comparing the individual scans from each instrument, we generally note remarkably good matches over even small millimeter-sized scan areas and between complex patterns of luminescence and PIXL P_2_O_5_ maps in the Thornton Gap, Berry Hollow (Fig. 6), and Dourbes (fig. S6) datasets, and little observation of structural mismatch between element maps and MCC images. We investigated any potential impact of x-ray point placement uncertainty on correlation results by comparing all versions of the same x-ray maps available to date and calculating statistics scores for all variations of the same target maps. Most of the x-ray target maps yielded no difference to correlation results. Newer versions of x-ray placements in Montpezat and Quartier target maps yielded a 0.1 to 0.3 positive increase in Pearson’s *r* and *SSIM* for the P_2_O_5_ and luminescence correlation. Therefore, the correlation results proved to be robust against PIXL x-ray placement uncertainty. The newest corrected x-ray maps to date were used for all figures and correlation statistics. Any potential further corrections of PIXL x-ray placements are predicted to improve the degree of luminescence and phosphorus correlation.

SHERLOC survey scans, detail scans, and PIXL elemental maps, respectively, have similar spatial resolutions, 0.144, 0.1, and 0.125 mm per scan point, respectively. Therefore, these maps were cross-correlated following processing steps described in fig. S9 using the following sequence: (i) MCC and ACI were coregistered and all PIXL maps were warped to fit ACI images; (ii) ACI, survey or detail grayscale maps, and warped PIXL grayscale maps were stacked and clipped manually to the largest rectangular overlap region; (iii) the pixel sizes of PIXL elemental maps were down-sampled to match the pixel sizes of SHERLOC maps using the pillow python module (*80*); (iv) the grayscale values of survey or detail grayscale luminescence maps were plotted against the grayscale values of all down-sampled PIXL elemental maps; and (v) image correlation statistics were calculated for all elemental and luminescence image pairs. Pearson’s *r* coefficient and *P* values were calculated using the Scipy python module (*70*), and structural similarity indexes *SSIM* were calculated using the scikit-image python module (*81*).

*SSIM* is a standard metric of similarity between two images (*82*), defined as

$$\text{SSIM}\left(x,y\right)=l\left(x,y\right)+c\left(x,y\right)+s\left(x,y\right)$$

Here, *x* and *y* are the two grayscale SHERLOC and PIXL element image values, *l* is a function of luminance, *c* is a function for contrast, and *s* is a function for structure. These functions are defined by the local mean, μ, SD, σ, and cross-covariance, σ*_xy_* through the following

$$l\left(x,y\right)=\frac{2\mu_{x}\mu_{y}+c_{1}\;}{\mu_{x}^{2}+\mu_{y}^{2}+c_{1}}$$

$$c\left(x,y\right)=\frac{2\sigma_{x}\sigma_{y}+c_{2}\;}{\sigma_{x}^{2}+\sigma_{y}^{2}+c_{2}}$$

$$s\left(x,y\right)=\frac{2\sigma_{\mathrm{xy}}+c_{3}\;}{\sigma_{x}^{2}\sigma_{y}^{2}+c_{3}}$$

where *c*_1_, *c*_2_, and *c*_3_ are constants. It follows that images with high similarity have *SSIM* close to 1 and images with low similarity have *SSIM* close to 0. Because this metric is sensitive to differences in brightness, a scalar used to adjust the 0 to 255 normalized grayscale values, we multiplied the brightness of all pixels in PIXL elemental maps by a single scalar to match the brightness of the SHERLOC luminescence intensity survey maps. In layman’s terms, PIXL elemental maps can be too dark or bright compared to the corresponding SHERLOC luminescence intensity survey maps. Hence, we multiplied all pixels in the maps by the same scalar to match the brightness of SHERLOC luminescence maps. This process does not affect calculated *r* or *P* values and is not applied when calculating *r* and *P* values. A range of different scalars was tested, and we chose the scalar where mean squared error between PIXL and SHERLOC maps was minimized, e.g., when the brightness of the two images was most similar.

The confidences of extraordinarily low *P* values and calculated *r* and *SSIM* values were assessed by calculating the distribution of index values obtained from a randomized dataset. This was done by calculating the *r* and *SSIM* index values of the images based on 10,000 random permutations of the SHERLOC luminescence image values and PIXL elemental maps. A 100% permutation here would mean a completely randomized image and a 10% permutation would have only 10% of the pixels moved around to a random position (figs. S10 and S11). If the PIXL and SHERLOC datasets are correlated, index values after 10% permutations should always have a higher index value than those after 100% permutation. We found that low *P* values are valid and that the positive *r* and *SSIM* cannot be produced by randomized datasets. We also found that according to expectations, 10% of permutations always score higher than fully randomized datasets.
