# Supplementary material for: Inorganic interpretation of luminescent materials encountered by the Perseverance rover on Mars
Source: Sci Adv. 2024 Sep 25;10(39):eadm8241. doi: 10.1126/sciadv.adm8241 (PMC11423895; doi:10.1126/sciadv.adm8241)

Science Advances
AAAS

Supplementary Materials for

Inorganic interpretation of luminescent materials encountered by the Perseverance rover on Mars

Eva L. Scheller *et al.*

Corresponding author: Eva L. Scheller, eschelle@mit.edu

Sci. Adv. **10**, eadm8241 (2024)
DOI: 10.1126/sciadv.adm8241

This PDF file includes:

Supplementary Text
Figs. S1 to S13
Tables S1 to S3

Supplementary Text

This document includes the following:

(1) Further description of laboratory spectra of NWA 10922 in section: Unmixing Ce3+ and organic contamination in Northwest Africa (NWA) 10922.

(2) Detailed file list for all data used in the manuscript in section: List of data files available on the PDS.

(3) Fig. S1 to S3 include further data analysis figures from NWA 10922.

(4) Fig. S4 to S13 and Table S1 to S3 include supplementary data analysis results and methodology figures from processing of the SHERLOC dataset mentioned in the main text.

<u>Unmixing Ce^{3+} and organic contamination in Northwest Africa (NWA) 10922</u>
In this section, we summarize further analyses performed to constrain the organic versus inorganic nature of luminescence in the NWA 10922 meteorite. All spectra taken from NWA 10922 contain a G-band related to terrestrial organic contamination (Fig. S2). This is expected due to terrestrial organic contamination. We discuss what luminescence features were found in NWA 10922 and whether these were organic or inorganic.

Phosphate grains and FTP clasts: Through the following series of arguments, we find clear evidence of inorganic 330-350 nm luminescence from Ce^{3+} in all phosphate grains measured in the meteorite. The main 330-350 nm broad luminescence from phosphate grains is resulting from Ce^{3+} and not terrestrial organic contamination through the following arguments: (1) The luminescence is markedly stronger when compared with luminescence from matrix, feldspars, and pyroxenes (Fig. S1). (2) The luminescence matches that of phosphate standards measured in (13). (3) The luminescence is also consistent with the expected two Ce^{3+} electron transition bands (5d→$^2F_{5/2}$, $^2F_{7/2}$ (4f)) that are expected for the Ca(I) site in apatite (39-40,49-50) (Fig. S2-S3). (4) Furthermore, the 330-350 nm luminescence intensity is linearly correlated with phosphate Raman peak intensity (Fig. 4D) and occurred when high phosphorous content was measured by XRF (Fig. 4A). 330-350 nm luminescence had no linear correlation with G-band intensity ($R^2 = 0.03$).

Given the asymmetry of the luminescence band, it is possible that there are additional bands ~450 nm (Fig. S3). Such a ~450 nm band would pose a degeneracy in the dataset due to the fact that macromolecular organic compounds, typical of terrestrial organic contamination, fluoresce in that wavelength (41), and the fact that a ~450 nm band is also expected for Ce^{3+} luminescence in the Ca(II) site in apatite (39). The intensity of the luminescence at 450 nm did not have a linear correlation with G-band intensity ($R^2 = 0.2$) or phosphate intensity ($R^2 = 0.1$). Hence, it cannot be resolved what is responsible for ~450 nm luminescence in phosphate grains,

although we prefer the interpretation that it is predominantly due to the terrestrial organic contamination (read below). There is also a minor unassigned 280 nm luminescence band, which appears similar to that observed in the Martian targets when low-intensity 280-290 nm luminescence and high-intensity 330-350 nm luminescence co-occurs.

Matrix, pyroxene, feldspar: Weak luminescence signals from matrix materials, pyroxenes, and feldspar are more difficult to constrain. We discuss possibilities for their origin in the below. Luminescence spectral profiles of matrix, plagioclase, and pyroxenes were largely similar and showed a band at both 330-350 nm and 410-430 nm (Fig. S2). The position of the 330-350 nm luminescence band is downshifted to ~330 nm. This is more typical of Ce^{3+} in monazite than in apatite (50) but can also occur in other minerals depending on the exact chemistry (e.g., Fig. 1). The 330-350 nm luminescence intensity showed no linear correlation to G-band intensity for any of these phases (R^2=0.08). Hence, we suggest that this less intense 330-350 nm luminescence is likely not related to terrestrial organic contamination but instead arises from small components of phosphates and/or other unresolved Ce^{3+}-containing minerals included in the matrix. The shift in wavelength position would suggest that the chemistry of the matrix Ce-bearing phase is different when compared with the phosphate grains (Fig. S2). In the case of measured pyroxene and plagioclase grains, we pose that the laser cannot avoid interaction with matrix phosphates when considering the ~75 um spot size. The 410-430 nm luminescence intensity showed a slight correlation with G-band intensity in both matrix, pyroxenes, and feldspar (R^2 = 0.4-0.5). Hence, we suggest that at least part of 410-430 nm luminescence is from terrestrial organic contamination like observations in (41).

<u>List of data files available on the PDS</u>
The Mars 2020 filename scheme is described in the EDR and RDR Software Interface Specification documents for PIXL and SHERLOC:
https://pds-geosciences.wustl.edu/m2020/urn-nasa-pds-mars2020_pixl/document/
https://pds-geosciences.wustl.edu/m2020/urn-nasa-pds-mars2020_sherloc/document/
Importantly, the filename product ID (characters 24-26) describe the content of the file. PIXL files with the RXL product ID contain X-ray beam location data. PIXL files with the RFS product ID contain the PIXL histograms. PIXL quantification results are located in PIXL files with the RQC product ID. SHERLOC files with the RLS product ID contain data on the SHERLOC laser spot locations. SHERLOC files with the ERA and ERB product IDs contain the raw active and dark frame spectral data. SHERLOC files with the EPA product ID contain the laser photodiode data used to conduct laser normalization. A full list of data files can be found in the supplement.

PIXL Data Files (https://pds-geosciences.wustl.edu/m2020/urn-nasa-pds-mars2020_pixl/):

Bellegarde

pe__0186_0683483744_000rxl__0070000699274310005___j07.csv

ps__0186_0683484569_000rfs__0070000699274310006___j02.csv
ps__0187_0683484569_000rqc__0070000699274310000___j01.csv

Montpezat

pe__0347_0697753955_000rxl__0092982122552837 0003___j06.csv
ps__0347_0697754703_000rfs__0092982122552837 0004___j04.csv
ps__0347_0697754703_000rqc__0092982122552837 0000___j01.csv
pe__0349_0697957194_000rxl__0092982123535879 0003___j05.csv
ps__0349_0697957949_000rfs__0092982123535879 0004___j02.csv
ps__0349_0697957949_000rqc__0092982123535879 0000___j01.csv

Alfalfa

pe__0369_0699718372_000rxl__0110108130744834 0003___j04.csv
ps__0369_0699719245_000rfs__0110108130744834 0004___j02.csv
ps__0369_0699719245_000rqc__0110108130744834 0000___j01.csv

Dourbes

pe__0257_0689790062_000rxl__0080000890639430006___j07.csv
ps__0257_0689790785_000rfs__0080000890639430007___j02.csv
ps__0257_0689790785_000rqc__0080000890639430000___j01.csv
pe__0269_0690860057_000rxl__0080000932582450003___j08.csv
ps__0269_0690861528_000rfs__0080000932582450004___j04.csv
ps__0270_0690861528_000rqc__0080000932582450000___j01.csv

Quartier

pe__0293_0692986008_000rxl__0090000101384711 0008___j06.csv
ps__0293_0692986818_000rfs__0090000101384711 0009___j02.csv
ps__0294_0692986818_000rqc__0090000101384711 0000___j01.csv
pe__0300_0693591971_000rxl__0090000104202753 0003___j04.csv
ps__0300_0693593437_000rfs__0090000104202753 0004___j02.csv
ps__0301_0693593437_000rqc__0090000104202753 0000___j01.csv

Thornton Gap

pe__0484_0709915608_000rxl__0261004168624645 0004___j06.csv
ps__0484_0709916480_000rfs__0261004168624645 0005___j02.csv
ps__0484_0709916378_000rqc__0261004168624645 0000___j01.csv
pe__0490_0710394319_000rxl__0261004170721793 0003___j04.csv
ps__0490_0710395021_000rfs__0261004170721793 0004___j02.csv
ps__0490_0710394904_000rqc__0261004170721793 0000___j01.csv

Berry Hollow

pe__0505_0711805127_000rxl__0261222176030213 0003___j04.csv
ps__0505_0711805999_000rfs__0261222176030213 0004___j02.csv
ps__0505_0711805893_000rqc__0261222176030213 0000___j01.csv
pe__0507_0711981970_000rxl__0261222176882177 0004___j03.csv
ps__0507_0711982738_000rfs__0261222176882177 0006___j03.csv

ps__0507_0711982637_000rqc__02612221768821770000___j01.csv

SHERLOC Data Files (https://pds-geosciences.wustl.edu/m2020/urn-nasa-pds-mars2020_sherloc):

Bellegarde

HDR 1:

SS__0186_0683476721_690EPA__0070000SRLC10500_108___J04.CSV
SS__0186_0683476721_690ERA__0070000SRLC10500_108___J04.CSV
SS__0186_0683476721_690ERB__0070000SRLC10500_108___J04.CSV
ss__0186_0683476721_690rls__0070000srlc10500_108___j04.csv

HDR 2:

SS__0186_0683477446_620EPA__0070000SRLC10500_208___J04.CSV
SS__0186_0683477446_620ERA__0070000SRLC10500_208___J04.CSV
SS__0186_0683477446_620ERB__0070000SRLC10500_208___J04.CSV
ss__0186_0683477446_620rls__0070000srlc10500_208___j04.csv

Survey:

SS__0186_0683478599_650EPA__0070000SRLC11420_108___J10.CSV
SS__0186_0683478599_650ERA__0070000SRLC11420_108___J10.CSV
SS__0186_0683478599_650ERB__0070000SRLC11420_108___J10.CSV
s__0186_0683478599_650rls__0070000srlc11420_108___j10.csv

Montpezat

HDR (part a):

SS__0349_0697951251_495EPA__0092982SRLC11360_108___J02.CSV
SS__0349_0697951251_495ERA__0092982SRLC11360_108___J02.CSV
SS__0349_0697951251_495ERB__0092982SRLC11360_108___J02.CSV
ss__0349_0697951251_495rls__0092982srlc11360_108___j02.csv

HDR (part b):

SS__0349_0697951900_355EPA__0092982SRLC11360_208___J02.CSV
SS__0349_0697951900_355ERA__0092982SRLC11360_208___J02.CSV
SS__0349_0697951900_355ERB__0092982SRLC11360_208___J02.CSV
ss__0349_0697951900_355rls__0092982srlc11360_208___j02.csv

survey:

SS__0349_0697952686_570EPA__0092982SRLC11420_108___J07.CSV
SS__0349_0697952686_570ERA__0092982SRLC11420_108___J07.CSV
SS__0349_0697952686_570ERB__0092982SRLC11420_108___J07.CSV
ss__0349_0697952686_570rls__0092982srlc11420_108___j07.csv

Alfalfa

HDR (part a):

SS__0370_0699813450_405EPA__0110108SRLC11360_108___J02.CSV
SS__0370_0699813450_405ERA__0110108SRLC11360_108___J02.CSV

SS__0370_0699813450_405ERB__0110108SRLC11360_108___J02.CSV
ss__0370_0699813450_405rls__0110108srlc11360_108___j02.csv

HDR (part b):

SS__0370_0699814099_215EPA__0110108SRLC11360_208___J02.CSV
SS__0370_0699814099_215ERA__0110108SRLC11360_208___J02.CSV
SS__0370_0699814099_215ERB__0110108SRLC11360_208___J02.CSV
ss__0370_0699814099_215rls__0110108srlc11360_208___j02.csv

survey:

SS__0370_0699814879_265EPA__0110108SRLC11420_108___J07.CSV
SS__0370_0699814879_265ERA__0110108SRLC11420_108___J07.CSV
SS__0370_0699814879_265ERB__0110108SRLC11420_108___J07.CSV
ss__0370_0699814879_265rls__0110108srlc11420_108___j07.csv

Garde

HDR 1 (part a):

SS__0207_0685346165_825EPA__0071836SRLC10501_108___J03.CSV
SS__0207_0685346165_825ERA__0071836SRLC10501_108___J03.CSV
SS__0207_0685346165_825ERB__0071836SRLC10501_108___J03.CSV
ss__0207_0685346165_825rls__0071836srlc10501_108___j03.csv

HDR 1 (part b):

SS__0207_0685346814_725EPA__0071836SRLC10501_208___J03.CSV
SS__0207_0685346814_725ERA__0071836SRLC10501_208___J03.CSV
SS__0207_0685346814_725ERB__0071836SRLC10501_208___J03.CSV
ss__0207_0685346814_725rls__0071836srlc10501_208___j03.csv

HDR 2 (part a):

SS__0207_0685347508_570EPA__0071836SRLC10501_108___J03.CSV
SS__0207_0685347508_570ERA__0071836SRLC10501_108___J03.CSV
SS__0207_0685347508_570ERB__0071836SRLC10501_108___J03.CSV
ss__0207_0685347508_570rls__0071836srlc10501_108___j03.csv

HDR 2 (part b):

SS__0207_0685348158_025EPA__0071836SRLC10501_208___J03.CSV
SS__0207_0685348158_025ERA__0071836SRLC10501_208___J03.CSV
SS__0207_0685348158_025ERB__0071836SRLC10501_208___J03.CSV
ss__0207_0685348158_025rls__0071836srlc10501_208___j03.csv

Survey:

SS__0207_0685348936_600EPA__0071836SRLC11421_108___J09.CSV
SS__0207_0685348936_600ERA__0071836SRLC11421_108___J09.CSV
SS__0207_0685348936_600ERB__0071836SRLC11421_108___J09.CSV
ss__0207_0685348936_600rls__0071836srlc11421_108___j09.csv

Detail 1 (part a):

SS__0208_0685432333_690EPA__0071836SRLC11370_108___J03.CSV

SS__0208_0685432333_690ERA__0071836SRLC11370_108___J03.CSV
SS__0208_0685432333_690ERB__0071836SRLC11370_108___J03.CSV
ss__0208_0685432333_690rls__0071836srlc11370_108___j03.csv

Detail 1 (part b):

SS__0208_0685432982_755EPA__0071836SRLC11370_208___J03.CSV
SS__0208_0685432982_755ERA__0071836SRLC11370_208___J03.CSV
SS__0208_0685432982_755ERB__0071836SRLC11370_208___J03.CSV
ss__0208_0685432982_755rls__0071836srlc11370_208___j03.csv

Detail 2 (part a):

SS__0208_0685433804_710EPA__0071836SRLC11371_108___J03.CSV
SS__0208_0685433804_710ERA__0071836SRLC11371_108___J03.CSV
SS__0208_0685433804_710ERB__0071836SRLC11371_108___J03.CSV
ss__0208_0685433804_710rls__0071836srlc11371_108___j03.csv

Detail 2 (part b):

SS__0208_0685434454_680EPA__0071836SRLC11371_208___J03.CSV
SS__0208_0685434454_680ERA__0071836SRLC11371_208___J03.CSV
SS__0208_0685434454_680ERB__0071836SRLC11371_208___J03.CSV
ss__0208_0685434454_680rls__0071836srlc11371_208___j03.csv

Detail 3 (part a):

SS__0208_0685435280_635EPA__0071836SRLC11372_108___J03.CSV
SS__0208_0685435280_635ERA__0071836SRLC11372_108___J03.CSV
SS__0208_0685435280_635ERB__0071836SRLC11372_108___J03.CSV
ss__0208_0685435280_635rls__0071836srlc11372_108___j03.csv

Detail 3 (part b):

SS__0208_0685435929_750EPA__0071836SRLC11372_208___J03.CSV
SS__0208_0685435929_750ERA__0071836SRLC11372_208___J03.CSV
SS__0208_0685435929_750ERB__0071836SRLC11372_208___J03.CSV
ss__0208_0685435929_750rls__0071836srlc11372_208___j03.csv

Dourbes

HDR (part a):

SS__0257_0689783904_065EPA__0080000SRLC11360_108___J03.CSV
SS__0257_0689783904_065ERA__0080000SRLC11360_108___J03.CSV
SS__0257_0689783904_065ERB__0080000SRLC11360_108___J03.CSV
ss__0257_0689783904_065rls__0080000srlc11360_108___j03.csv

HDR (part b):

SS__0257_0689784553_585EPA__0080000SRLC11360_208___J03.CSV
SS__0257_0689784553_585ERA__0080000SRLC11360_208___J03.CSV
SS__0257_0689784553_585ERB__0080000SRLC11360_208___J03.CSV
ss__0257_0689784553_585rls__0080000srlc11360_208___j03.csv

Survey 1:

SS__0257_0689785335_515EPA__0080000SRLC11421_108___J06.CSV
SS__0257_0689785335_515ERA__0080000SRLC11421_108___J06.CSV
SS__0257_0689785335_515ERB__0080000SRLC11421_108___J06.CSV
ss__0257_0689785335_515rls__0080000srlc11421_108___j06.csv

Survey 2:
SS__0269_0690848406_825EPA__0080000SRLC11421_108___J06.CSV
SS__0269_0690848406_825ERA__0080000SRLC11421_108___J06.CSV
SS__0269_0690848406_825ERB__0080000SRLC11421_108___J06.CSV
ss__0269_0690848406_825rls__0080000srlc11421_108___j06.csv

Detail 1 (part a):
SS__0269_0690849690_630EPA__0080000SRLC11370_108___J04.CSV
SS__0269_0690849690_630ERA__0080000SRLC11370_108___J04.CSV
SS__0269_0690849690_630ERB__0080000SRLC11370_108___J04.CSV
ss__0269_0690849690_630rls__0080000srlc11370_108___j04.csv

Detail 1 (part b):
SS__0269_0690850340_585EPA__0080000SRLC11370_208___J04.CSV
SS__0269_0690850340_585ERA__0080000SRLC11370_208___J04.CSV
SS__0269_0690850340_585ERB__0080000SRLC11370_208___J04.CSV
ss__0269_0690850340_585rls__0080000srlc11370_208___j04.csv

Detail 2 (part a):
SS__0269_0690851164_255EPA__0080000SRLC11373_108___J04.CSV
SS__0269_0690851164_255ERA__0080000SRLC11373_108___J04.CSV
SS__0269_0690851164_255ERB__0080000SRLC11373_108___J04.CSV
ss__0269_0690851164_255rls__0080000srlc11373_108___j04.csv

Detail 2 (part b):
SS__0269_0690851813_405EPA__0080000SRLC11373_208___J04.CSV
SS__0269_0690851813_405ERA__0080000SRLC11373_208___J04.CSV
SS__0269_0690851813_405ERB__0080000SRLC11373_208___J04.CSV
ss__0269_0690851813_405rls__0080000srlc11373_208___j04.csv

Detail 3 (part a):
SS__0269_0690852635_600EPA__0080000SRLC11374_108___J04.CSV
SS__0269_0690852635_600ERA__0080000SRLC11374_108___J04.CSV
SS__0269_0690852635_600ERB__0080000SRLC11374_108___J04.CSV
ss__0269_0690852635_600rls__0080000srlc11374_108___j04.csv

Detail 3 (part b):
SS__0269_0690853284_920EPA__0080000SRLC11374_208___J03.CSV
SS__0269_0690853284_920ERA__0080000SRLC11374_208___J03.CSV
SS__0269_0690853284_920ERB__0080000SRLC11374_208___J03.CSV
ss__0269_0690853284_920rls__0080000srlc11374_208___j03.csv

Quartier

HDR (part a):
SS__0293_0692979640_115EPA__0090000SRLC11360_108___J03.CSV
SS__0293_0692979640_115ERA__0090000SRLC11360_108___J03.CSV
SS__0293_0692979640_115ERB__0090000SRLC11360_108___J03.CSV
ss__0293_0692979640_115rls__0090000srlc11360_108___j03.csv
HDR (part b):
SS__0293_0692980289_265EPA__0090000SRLC11360_208___J03.CSV
SS__0293_0692980289_265ERA__0090000SRLC11360_208___J03.CSV
SS__0293_0692980289_265ERB__0090000SRLC11360_208___J03.CSV
ss__0293_0692980289_265rls__0090000srlc11360_208___j03.csv
Survey 1:
SS__0293_0692981070_175EPA__0090000SRLC11420_108___J06.CSV
SS__0293_0692981070_175ERA__0090000SRLC11420_108___J06.CSV
SS__0293_0692981070_175ERB__0090000SRLC11420_108___J06.CSV
ss__0293_0692981070_175rls__0090000srlc11420_108___j06.csv
Survey 2:
SS__0304_0693959951_875EPA__0090000SRLC11451_108___J05.CSV
SS__0304_0693959951_875ERA__0090000SRLC11451_108___J05.CSV
SS__0304_0693959951_875ERB__0090000SRLC11451_108___J05.CSV
ss__0304_0693959951_875rls__0090000srlc11451_108___j05.csv
Detail 1 (part a):
SS__0304_0693961008_675EPA__0090000SRLC11372_108___J02.CSV
SS__0304_0693961008_675ERA__0090000SRLC11372_108___J02.CSV
SS__0304_0693961008_675ERB__0090000SRLC11372_108___J02.CSV
ss__0304_0693961008_675rls__0090000srlc11372_108___j02.csv
Detail 1 (part b):
SS__0304_0693961658_010EPA__0090000SRLC11372_208___J02.CSV
SS__0304_0693961658_010ERA__0090000SRLC11372_208___J02.CSV
SS__0304_0693961658_010ERB__0090000SRLC11372_208___J02.CSV
ss__0304_0693961658_010rls__0090000srlc11372_208___j02.csv
Detail 2 (part a):
SS__0304_0693962475_780EPA__0090000SRLC11373_108___J02.CSV
SS__0304_0693962475_780ERA__0090000SRLC11373_108___J02.CSV
SS__0304_0693962475_780ERB__0090000SRLC11373_108___J02.CSV
ss__0304_0693962475_780rls__0090000srlc11373_108___j02.csv
Detail 2 (part b):
SS__0304_0693963125_235EPA__0090000SRLC11373_208___J02.CSV
SS__0304_0693963125_235ERA__0090000SRLC11373_208___J02.CSV
SS__0304_0693963125_235ERB__0090000SRLC11373_208___J02.CSV
ss__0304_0693963125_235rls__0090000srlc11373_208___j02.csv

Detail 3 (part a):
SS__0304_0693963943_980EPA__0090000SRLC11374_108___J02.CSV
SS__0304_0693963943_980ERA__0090000SRLC11374_108___J02.CSV
SS__0304_0693963943_980ERB__0090000SRLC11374_108___J02.CSV
ss__0304_0693963943_980rls__0090000srlc11374_108___j02.csv
Detail 3 (part b):
SS__0304_0693964592_870EPA__0090000SRLC11374_208___J02.CSV
SS__0304_0693964592_870ERA__0090000SRLC11374_208___J02.CSV
SS__0304_0693964592_870ERB__0090000SRLC11374_208___J02.CSV
ss__0304_0693964592_870rls__0090000srlc11374_208___j02.csv
Detail 4 (part a):
SS__0304_0693965408_820EPA__0090000SRLC11375_108___J02.CSV
SS__0304_0693965408_820ERA__0090000SRLC11375_108___J02.CSV
SS__0304_0693965408_820ERB__0090000SRLC11375_108___J02.CSV
ss__0304_0693965408_820rls__0090000srlc11375_108___j02.csv
Detail 4 (part b):
SS__0304_0693966058_170EPA__0090000SRLC11375_208___J02.CSV
SS__0304_0693966058_170ERA__0090000SRLC11375_208___J02.CSV
SS__0304_0693966058_170ERB__0090000SRLC11375_208___J02.CSV
ss__0304_0693966058_170rls__0090000srlc11375_208___j02.csv

Thornton Gap

HDR 1 (part a):
SS__0489_0710377627_500EPA__0261004SRLC11360_108___J02.CSV
SS__0489_0710377627_500ERA__0261004SRLC11360_108___J02.CSV
SS__0489_0710377627_500ERB__0261004SRLC11360_108___J02.CSV
ss__0489_0710377627_500rls__0261004srlc11360_108___j02.csv
HDR 1 (part b):
SS__0489_0710378276_295EPA__0261004SRLC11360_208___J02.CSV
SS__0489_0710378276_295ERA__0261004SRLC11360_208___J02.CSV
SS__0489_0710378276_295ERB__0261004SRLC11360_208___J02.CSV
ss__0489_0710378276_295rls__0261004srlc11360_208___j02.csv
Detail 1 (part a):
SS__0489_0710379058_350EPA__0261004SRLC11370_108___J02.CSV
SS__0489_0710379058_350ERA__0261004SRLC11370_108___J02.CSV
SS__0489_0710379058_350ERB__0261004SRLC11370_108___J02.CSV
ss__0489_0710379058_350rls__0261004srlc11370_108___j02.csv
Detail 1 (part b):
SS__0489_0710379707_400EPA__0261004SRLC11370_208___J02.CSV
SS__0489_0710379707_400ERA__0261004SRLC11370_208___J02.CSV
SS__0489_0710379707_400ERB__0261004SRLC11370_208___J02.CSV

ss__0489_0710379707_400rls__0261004srlc11370_208___j03.csv
Survey 1:
SS__0489_0710380488_510EPA__0261004SRLC11420_108___J03.CSV
SS__0489_0710380488_510ERA__0261004SRLC11420_108___J03.CSV
SS__0489_0710380488_510ERB__0261004SRLC11420_108___J03.CSV
ss__0489_0710380488_510rls__0261004srlc11420_108___j03.csv
HDR 2 (part a):
SS__0489_0710382169_545EPA__0261004SRLC11360_108___J02.CSV
SS__0489_0710382169_545ERA__0261004SRLC11360_108___J02.CSV
SS__0489_0710382169_545ERB__0261004SRLC11360_108___J02.CSV
ss__0489_0710382169_545rls__0261004srlc11360_108___j02.csv
HDR 2 (part b):
SS__0489_0710382818_445EPA__0261004SRLC11360_208___J02.CSV
SS__0489_0710382818_445ERA__0261004SRLC11360_208___J02.CSV
SS__0489_0710382818_445ERB__0261004SRLC11360_208___J02.CSV
ss__0489_0710382818_445rls__0261004srlc11360_208___j02.csv
Detail 2 (part a):
SS__0489_0710383600_515EPA__0261004SRLC11370_108___J02.CSV
SS__0489_0710383600_515ERA__0261004SRLC11370_108___J02.CSV
SS__0489_0710383600_515ERB__0261004SRLC11370_108___J02.CSV
ss__0489_0710383600_515rls__0261004srlc11370_108___j03.csv
Detail 2 (part b):
SS__0489_0710384249_650EPA__0261004SRLC11370_208___J02.CSV
SS__0489_0710384249_650ERA__0261004SRLC11370_208___J02.CSV
SS__0489_0710384249_650ERB__0261004SRLC11370_208___J02.CSV
ss__0489_0710384249_650rls__0261004srlc11370_208___j02.csv
Survey 2:
SS__0489_0710385032_565EPA__0261004SRLC11420_108___J03.CSV
SS__0489_0710385032_565ERA__0261004SRLC11420_108___J03.CSV
SS__0489_0710385032_565ERB__0261004SRLC11420_108___J03.CSV
ss__0489_0710385032_565rls__0261004srlc11420_108___j03.csv

Berry Hollow

HDR (part a):
SS__0505_0711795172_280EPA__0261222SRLC11360_108___J02.CSV
SS__0505_0711795172_280ERA__0261222SRLC11360_108___J02.CSV
SS__0505_0711795172_280ERB__0261222SRLC11360_108___J02.CSV
ss__0505_0711795172_280rls__0261222srlc11360_108___j02.csv
HDR (part b):
SS__0505_0711795820_880EPA__0261222SRLC11360_208___J02.CSV
SS__0505_0711795820_880ERA__0261222SRLC11360_208___J02.CSV

SS__0505_0711795820_880ERB__0261222SRLC11360_208___J02.CSV
ss__0505_0711795820_880rls__0261222srlc11360_208___j02.csv

Detail 1 (part a):

SS__0505_0711796602_020EPA__0261222SRLC11370_108___J02.CSV
SS__0505_0711796602_020ERA__0261222SRLC11370_108___J02.CSV
SS__0505_0711796602_020ERB__0261222SRLC11370_108___J02.CSV
ss__0505_0711796602_020rls__0261222srlc11370_108___j02.csv

Detail 1 (part b):

SS__0505_0711797251_025EPA__0261222SRLC11370_208___J02.CSV
SS__0505_0711797251_025ERA__0261222SRLC11370_208___J02.CSV
SS__0505_0711797251_025ERB__0261222SRLC11370_208___J02.CSV
ss__0505_0711797251_025rls__0261222srlc11370_208___j02.csv

Survey:

SS__0513_0712513080_580EPA__0261222SRLC11440_108___J02.CSV
SS__0513_0712513080_580ERA__0261222SRLC11440_108___J02.CSV
SS__0513_0712513080_580ERB__0261222SRLC11440_108___J02.CSV
ss__0513_0712513080_580rls__0261222srlc11440_108___j02.csv

Detail 1 (part a):

SS__0513_0712514114_395EPA__0261222SRLC11372_108___J02.CSV
SS__0513_0712514114_395ERA__0261222SRLC11372_108___J02.CSV
SS__0513_0712514114_395ERB__0261222SRLC11372_108___J02.CSV
ss__0513_0712514114_395rls__0261222srlc11372_108___j02.csv

Detail 1 (part b):

SS__0513_0712514763_385EPA__0261222SRLC11372_208___J02.CSV
SS__0513_0712514763_385ERA__0261222SRLC11372_208___J02.CSV
SS__0513_0712514763_385ERB__0261222SRLC11372_208___J02.CSV
ss__0513_0712514763_385rls__0261222srlc11372_208___j02.csv

Detail 2 (part a):

SS__0513_0712515579_390EPA__0261222SRLC11373_108___J02.CSV
SS__0513_0712515579_390ERA__0261222SRLC11373_108___J02.CSV
SS__0513_0712515579_390ERB__0261222SRLC11373_108___J02.CSV
ss__0513_0712515579_390rls__0261222srlc11373_108___j02.csv

Detail 2 (part b):

SS__0513_0712516228_405EPA__0261222SRLC11373_208___J02.CSV
SS__0513_0712516228_405ERA__0261222SRLC11373_208___J02.CSV
SS__0513_0712516228_405ERB__0261222SRLC11373_208___J02.CSV
ss__0513_0712516228_405rls__0261222srlc11373_208___j02.csv

Detail 3 (part a):

SS__0513_0712517045_550EPA__0261222SRLC11374_108___J02.CSV
SS__0513_0712517045_550ERA__0261222SRLC11374_108___J02.CSV

SS__0513_0712517045_550ERB__0261222SRLC11374_108___J02.CSV
ss__0513_0712517045_550rls__0261222srlc11374_108___j02.csv

Detail 3 (part b):

SS__0513_0712517694_430EPA__0261222SRLC11374_208___J02.CSV
SS__0513_0712517694_430ERA__0261222SRLC11374_208___J02.CSV
SS__0513_0712517694_430ERB__0261222SRLC11374_208___J02.CSV
ss__0513_0712517694_430rls__0261222srlc11374_208___j02.csv

Detail 4 (part a):

SS__0513_0712518509_495EPA__0261222SRLC11375_108___J02.CSV
SS__0513_0712518509_495ERA__0261222SRLC11375_108___J02.CSV
SS__0513_0712518509_495ERB__0261222SRLC11375_108___J02.CSV
ss__0513_0712518509_495rls__0261222srlc11375_108___j02.csv

Detail 4 (part b):

SS__0513_0712519158_660EPA__0261222SRLC11375_208___J02.CSV
SS__0513_0712519158_660ERA__0261222SRLC11375_208___J02.CSV
SS__0513_0712519158_660ERB__0261222SRLC11375_208___J02.CSV
ss__0513_0712519158_660rls__0261222srlc11375_208___j02.csv

Table S1. Bulk composition of rock targets from PIXL compared to number (n) of luminescence features found in SHERLOC survey and HDR scans (see section 2 for definitions). We track the bulk P_2O_5 wt% as relevant proxies for phosphate contents. We track SiO_2 wt% as a proxy for more high-Si phases that may contain silica or silicate defects. We track K_2O wt% as a proxy for more felsic compositions. We can track FeO_T wt% as a proxy for phases that would be strongly UV absorbing and therefore lead to false non-detections.

**Target**	**Lithology**	**SiO_2 (wt%)**	**FeO_T (wt%)**	**P_2O_5 (wt%)**	**K_2O (wt%)**	**$n_{330-350}$ *survey***	**$n_{330-350}$ *HDR***	**$n_{330-350}$ *detail***	**$n_{270-290}$ *survey***	**$n_{270-290}$ *HDR***	**$n_{270-290}$ *detail***
Bellegarde	Basalt	43.8±2.2	23.3±1.2	2.8±0.6	1.1±0.3	227	57	N/A	55	25	N/A
Alfalfa	Basalt	56.1±2.8	11.1±0.6	1.0±0.3	1.9±0.5	111	78	N/A	90	58	N/A
Montpezat	Basalt	44.8±2.2	18.3±0.9	1.2±0.4	0.8±0.3	55	51	N/A	9	12	N/A
Dourbes scan 1	Olivine cumulate	39.4±2.0	29.3±1.6	0.3±0.2	0.1±0.1	23	8	N/A	0	4	N/A
Dourbes scan 2	Olivine cumulate	37.2±1.9	29.3±1.5	0.8±0.3	0.3±0.2	47	N/A	48	1	N/A	3
Quartier	Olivine cumulate	33.3±1.7	29.4±1.5	0.4±0.2	0.1±0.1	22	2	13	0	0	2
Thornton Gap	Lower Delta Sediment	22.8±1.1	23.8±1.2	0.3±0.2	0.3±0.2	4	2	13	0	4	0
Berry Hollow	Lower Delta Sediment	32.2±1.6	17.6±0.9	0.8±0.3	0.2±0.2	1	1	N/A	0	0	0

N/A means that an HDR or detail dataset was not obtained for that scan.

Fig. S1. Example of gentle positive slope luminescence expected from macromolecular carbon co-occurring with Raman G-band in the meteorite caltarget, SaU008, measured on SHERLOC. These organic compounds occur in calcite crystals, suggesting that they arise from terrestrial contamination.

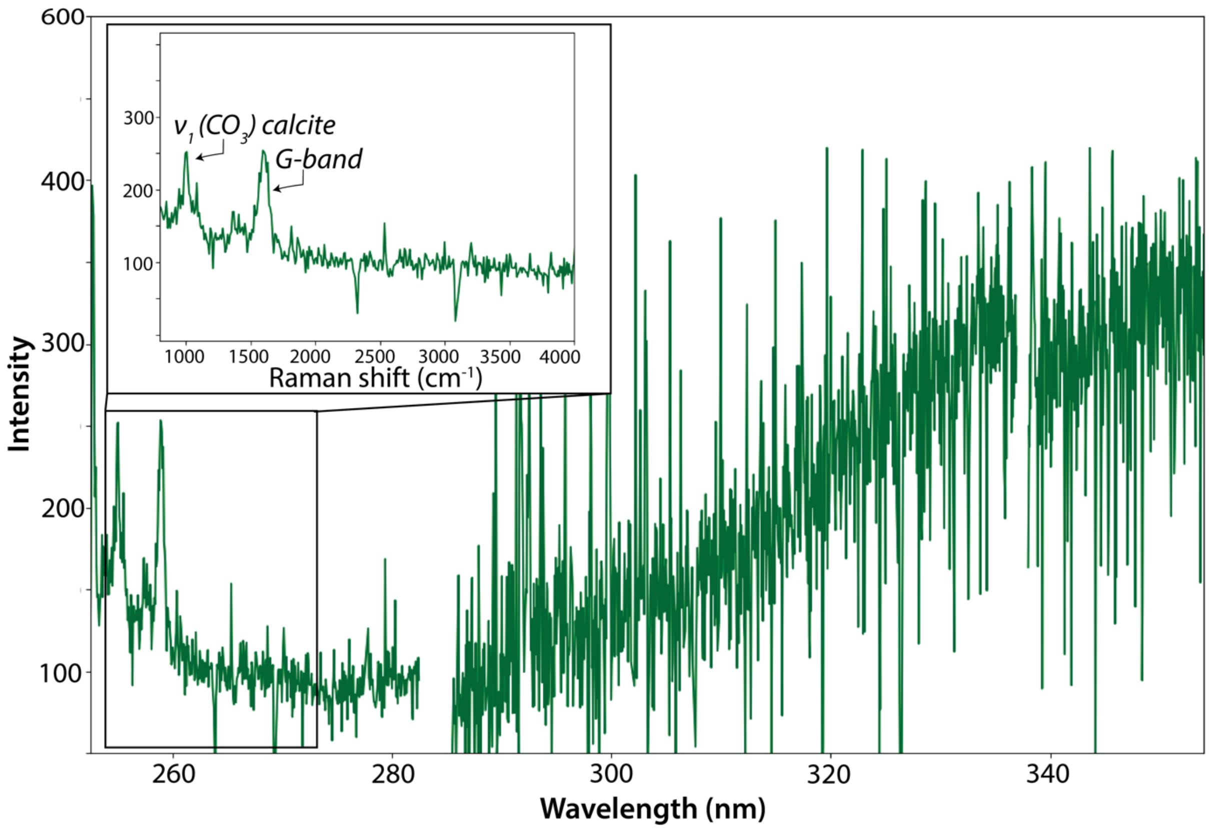

Fig. S2. Raman and luminescence spectra of phosphate grains compared with Raman and luminescence spectra of matrix (wherein sub-micron µm phosphate grains are common), feldspar grains, and pyroxene grains within NWA 10922. Spot numbers refer to locations in the XRF image from Fig. 4A.

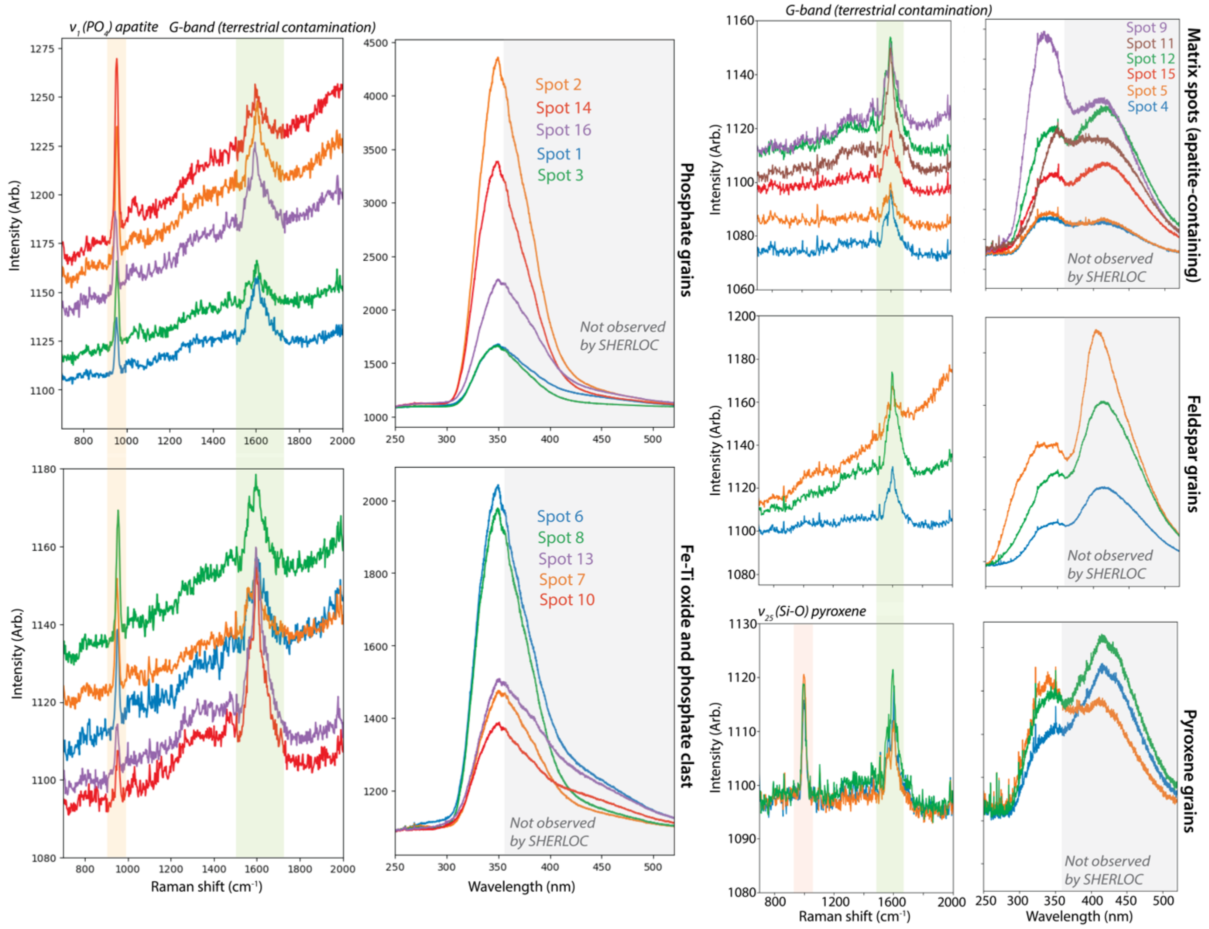

Fig. S3. Example of Gaussian modeling utilized for three meteorite measurements shown in Fig. S2 and Fig. 4. Spot 2 and spot 14 are from phosphate grains and exhibit a high intensity 330-350 nm signal matching the expected Ce^{3+} transitions. Spot 9 is from the matrix region. We note that a doublet is present at 330-350 nm but the spectral positions and intensity ratios change. Note much lower intensity signal in spot 9 in comparison to the phosphate grains. This allows the ~430 nm band to be observed directly in the data. We prefer assignment of ~430 nm band to macromolecular carbon terrestrial contamination that is omnipresent in the meteorite. This figure exemplifies gaussian mixture modeling methodology used for all experiment and SHERLOC flight data for investigation of spectral positions and shapes.

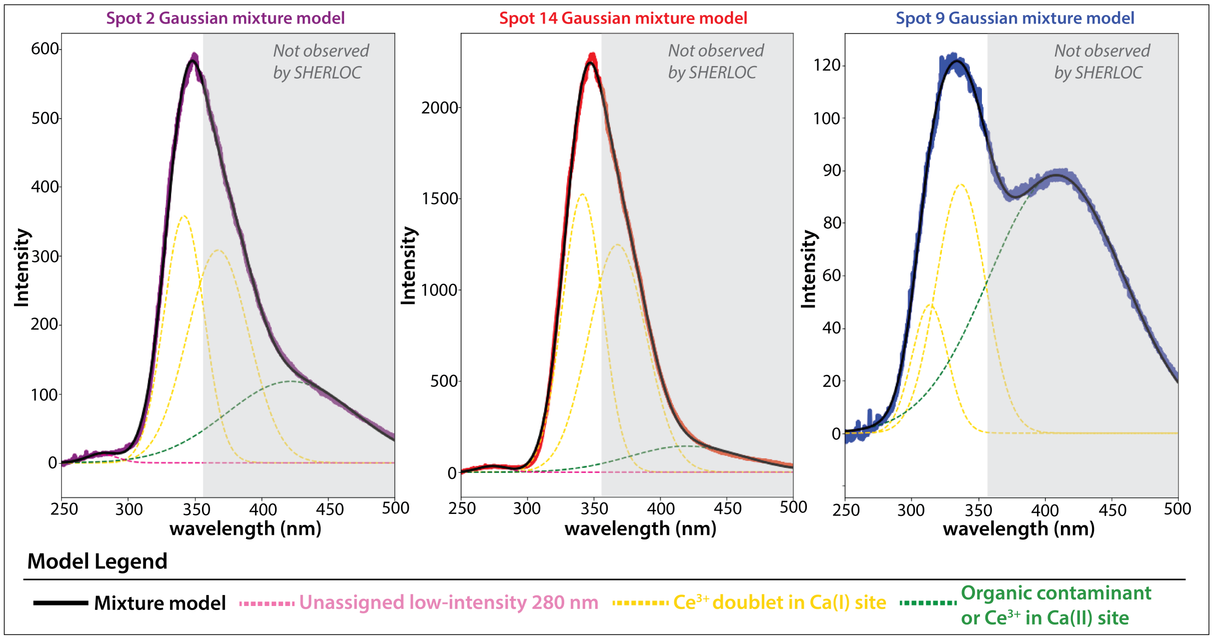

Fig. S4. Histogram of peak positions of analyzed 950-970 cm^{-1} Raman peaks from SHERLOC data across all scans. All peak positions have on average ±5 cm^{-1} uncertainty. Grey bars show the spread in SHERLOC Raman peak positions across scans. Orange and red bars show the spread in Raman peak positions of chlorapatites and hydroxy/fluorapatite, respectively, measured on the ACRONM laboratory instrument.

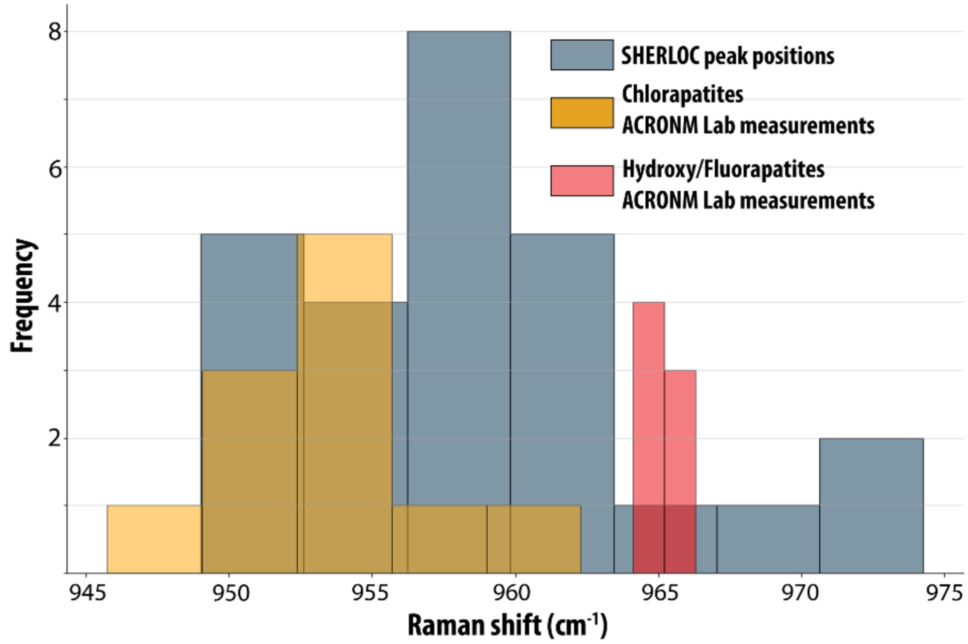

Fig. S5: Left panels show luminescence identifications (stars) superposed on Raman peak identifications (circles) in analyzed HDR scans from rock targets in the Máaz fm. Open white circles indicate no detections. Right panels show 330-350 nm luminescence (and coinciding 270-290 nm luminescence) superposed on co-registered P_2O_5 PIXL maps; only features that coincide with the PIXL map are displayed. As the co-registration between maps have inherent uncertainty and the spacing of SHERLOC HDR grid points are so far apart compared to the spacing of PIXL spots, the datasets cannot be quantitatively compared. However, we note that the HDR grid scans are just a down-sampled version of survey scans shown in Fig. 5 as they cover the same area in the rock. Spectra in these HDR scans that do not obviously correlate with phosphate or P_2O_5 were explored in Fig. S11.

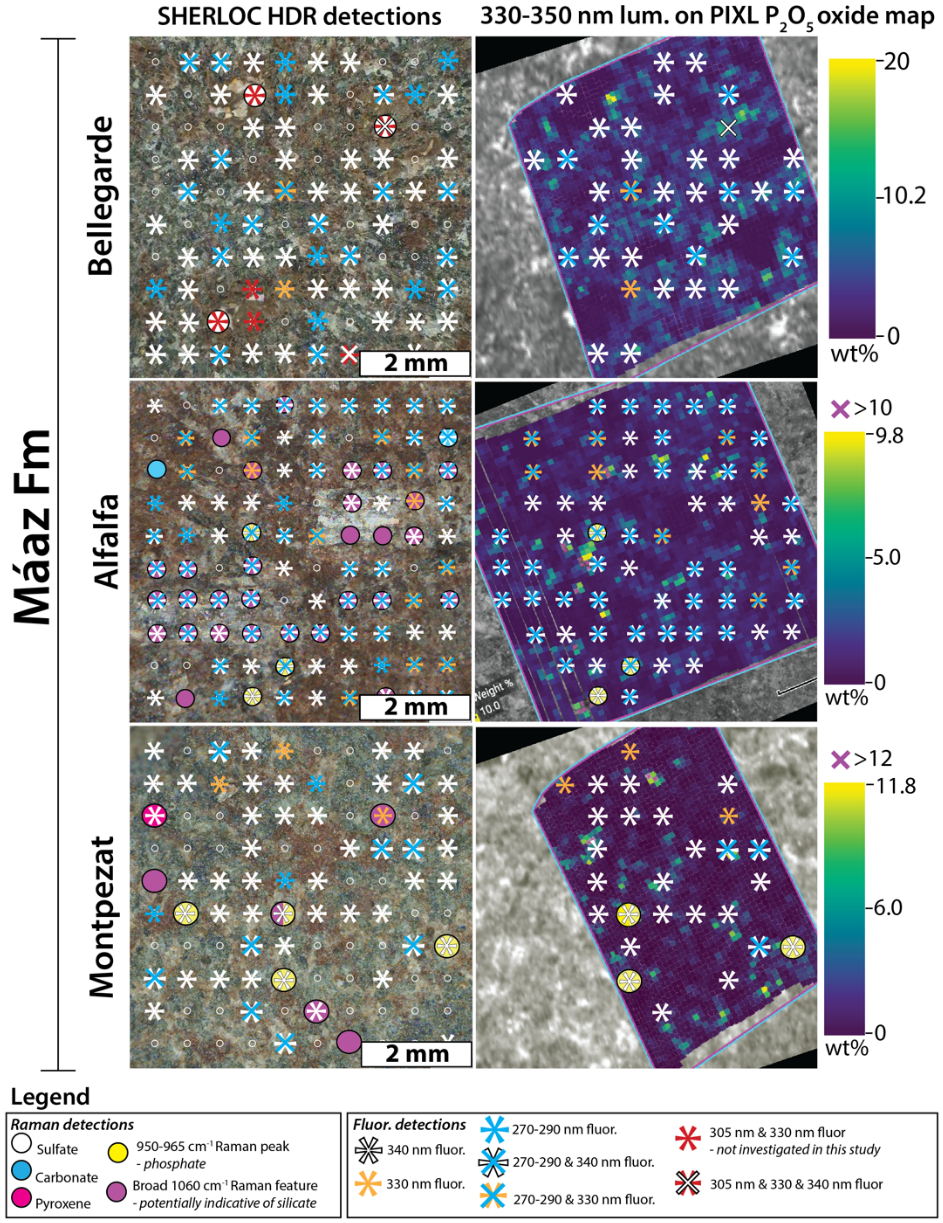

Fig. S6: Left panels show luminescence identifications (stars) superposed on Raman peak identifications (circles) in analyzed HDR scans from rock targets in the Máaz fm. Open white circles indicate no detections. Right panels show 270-290 nm luminescence (and coinciding 330-350 nm luminescence) superposed on co-registered PIXL SiO_2, Al_2O_3, and FeO_T composite maps; only features that coincide with the PIXL map are displayed. Correlations are not easily explored with the HDR scans where the grid spacing is on a different scale compared to PIXL maps, however, HDR detections of 270-290 nm luminescence are found with high-Si phases as well as a variety of silicate compositions not explored at depth in this study.

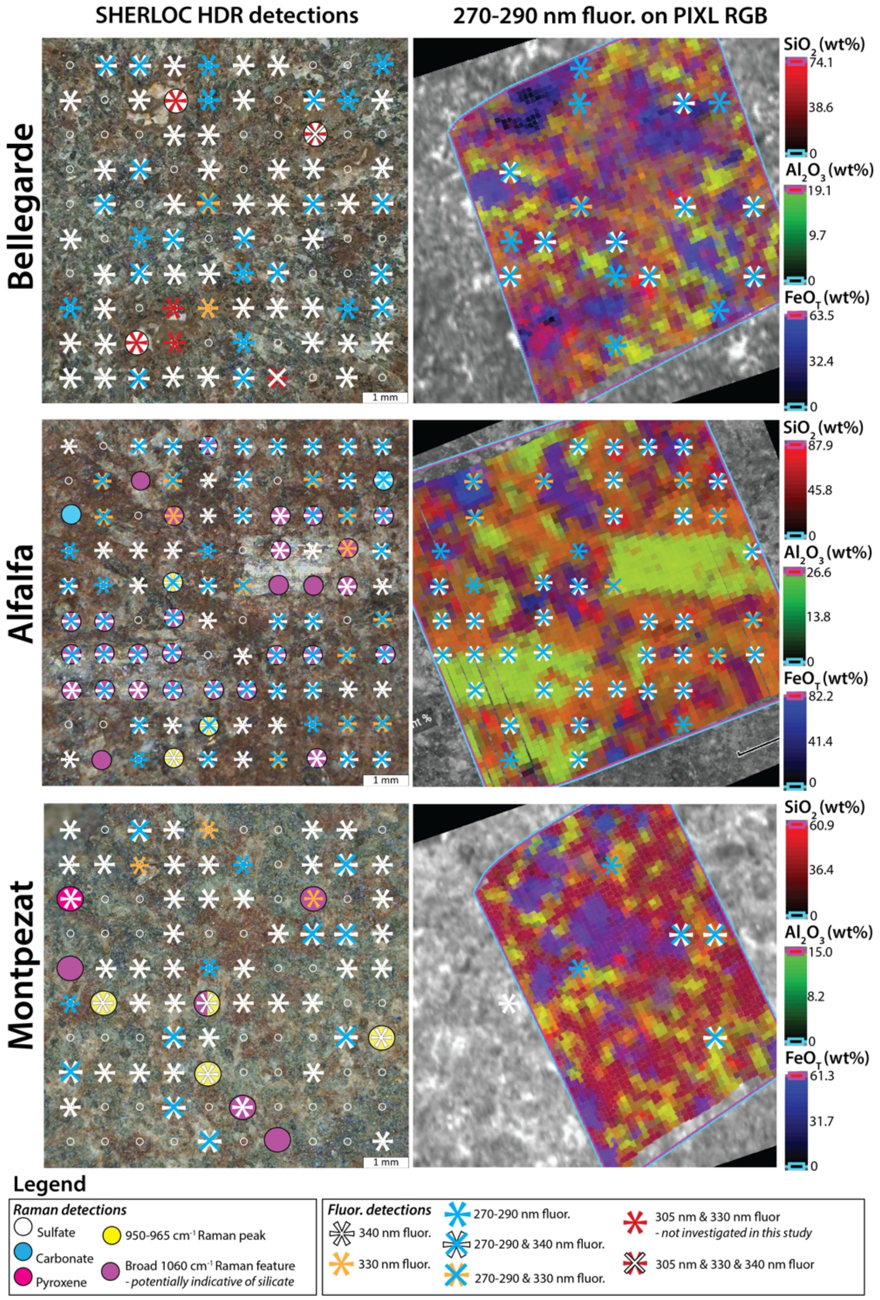

Fig. S7. Data from detail scans of the Dourbes scan 2 abraded rock in the Séítah fm. Top panel shows Raman and luminescence detections are symbolized with colored circles and stars respectively on colorized ACI-WATSON image merge. Open white circles indicate that no Raman features were detected. Middle panel shows the 330-350 nm luminescence detection locations on co-registered PIXL P_2O_5 wt% map. Lowermost panel shows luminescence heatmap processed through the same methodology as Fig. 5-6. High intensity luminescence occurs with the highest P_2O_5 wt%. Note the spatial association between P_2O_5 and both 340-350 nm and 330 nm luminescence, despite a lack of phosphate Raman peak identifications. Spectra in these detail scans that do not obviously correlate with phosphate or P_2O_5 were explored in Fig. S11.

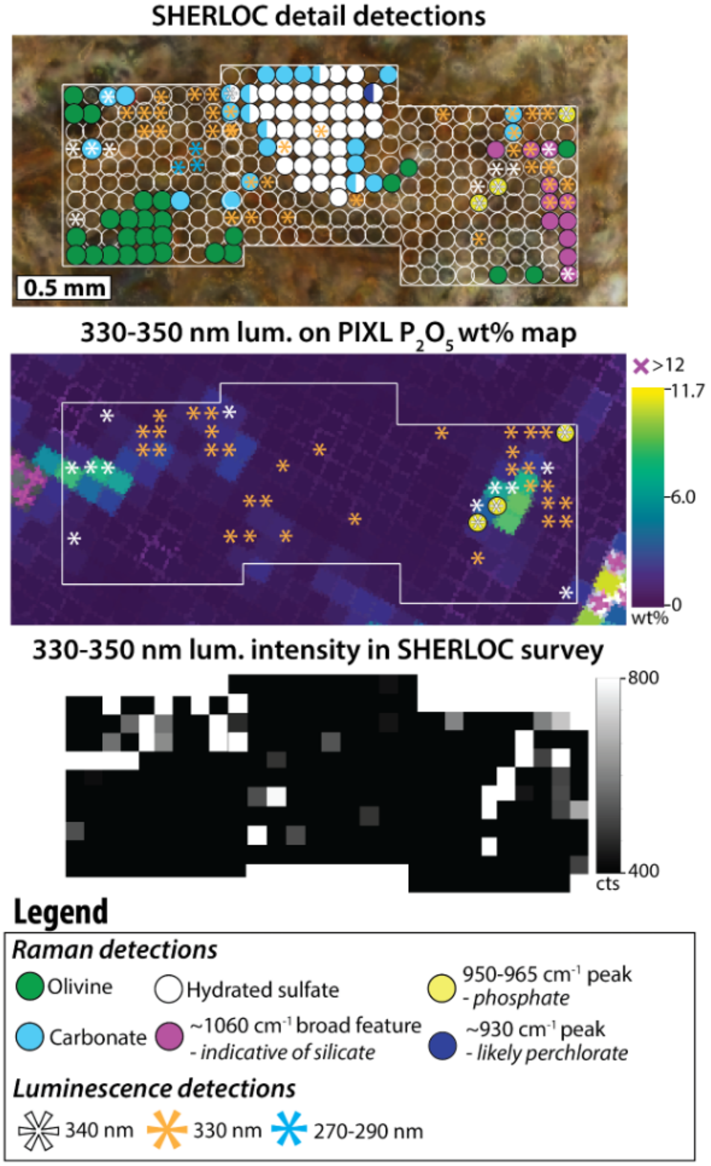

Fig. S8: Data from detail scans of the Garde abraded rock in the Séítah fm. Here, no PIXL data was obtained for this abraded rock. Left panels show colorized ACI-WATSON image merges. Middle panels show Raman and luminescence detections symbolized with colored circles and stars. Right panels show greyscale heatmaps of 330-350 nm luminescence and 270-290 nm luminescence with same processed through the same methodology as Figs. 5-6. Note that the correlation between 330-350 nm luminescence, 270-290 nm luminescence, and Raman identifications of phosphate are unusually strong in this scan.

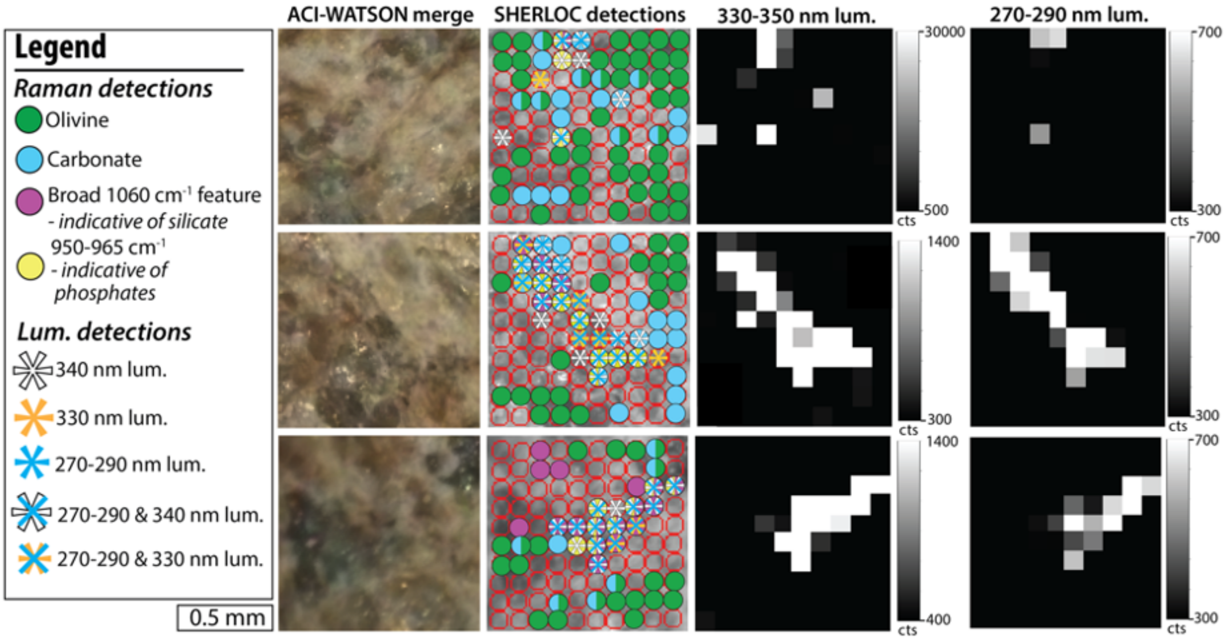

Fig. S9: Processing methodology to co-register and examine cross-correlation of PIXL elemental maps with SHERLOC luminescence maps. The ENVI software correlation method is used for all results and figures. However, the correlation statistics methodology is only used for comparisons between SHERLOC survey luminescence maps and PIXL elemental maps where the grid spacing and amount of data points are sufficient for quantitative correlations.

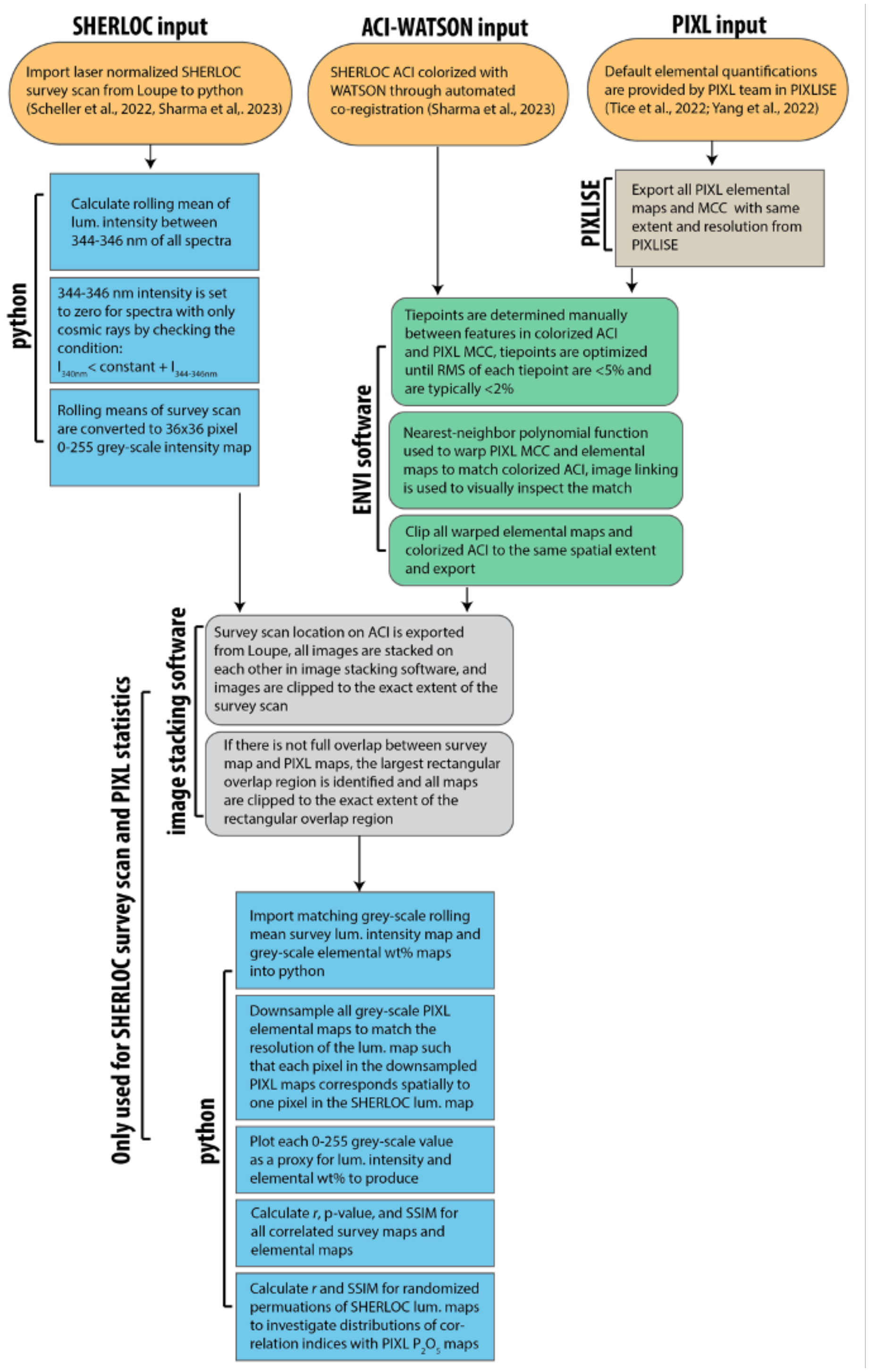

Table S2. Summary of correlation statistics between all other PIXL elemental maps and SHERLOC 330-350 nm luminescence intensity maps following same methods as in Table 3. We evaluate the same statistical metrics, *r, p*-value, and *SSIM*, for 330-350 nm luminescence intensity correlations to all PIXL elemental wt% maps. When *r* is 0.4 or *r* is -0.4 and the associated *p*-value is <10^{-3}, we indicate that there is a correlation as strong as the weakest P_2O_5 correlation (in the Alfalfa target).

**Target**	**r**	**p-value**	**SSIM**	**Correlation**
Bellegarde				
SiO_2	0.1	10^{-2}	0	None
Al_2O_3	0	10^{-1}	0	None
CaO	0.2	10^{-15}	0.2	None
Na_2O	0	10^{-1}	0.2	None
K_2O	0.1	10^{-5}	0.1	None
FeO_T	-0.2	10^{-7}	-0.1	None
MgO	-0.1	10^{-2}	0	None
SO_3	-0.2	10^{-7}	0	None
Cl	0	10^{-1}	0	None
Alfalfa				
SiO_2	0	10^{-1}	0	None
Al_2O_3	-0.1	10^{-3}	0	None
CaO	0	10^{-1}	-0.1	None
Na_2O	-0.1	10^{-3}	0	None
K_2O	0.1	10^{-3}	0.1	None
FeO_T	0	10^{-1}	0.1	None
MgO	0	10^{-1}	0.1	None
SO_3	0	10^{-1}	0	None
Cl	0.1	10^{-2}	0.1	None
Montpezat				
SiO_2	0.1	10^{-1}	0	None

Al_2O_3	0.2	10^{-4}	0.1	None
CaO	0.1	10^{-1}	0	None
Na_2O	0.2	10^{-5}	0.1	None
K_2O	0.2	10^{-3}	0.2	None
FeO_T	-0.1	10^{-2}	0	None
MgO	-0.2	10^{-7}	-0.1	None
SO_3	0.1	10^{-1}	0.2	None
Cl	0	1	0	None
Dourbes Scan 1				
SiO_2	0.1	10^{-4}	0	None
**Al_2O_3**	**0.4**	**10^{-23}**	**0.4**	**Positive**
CaO	0.1	10^{-2}	0.3	None
Na_2O	0.3	10^{-17}	0.5	None
**K_2O**	**0.4**	**10^{-23}**	**0.5**	**Positive**
FeO_T	-0.2	10^{-8}	-0.1	None
MgO	-0.3	10^{-15}	-0.1	None
SO_3	0.1	10^{-1}	0.5	None
Cl	0	10^{-1}	0.3	None
Dourbes Scan 2				
SiO_2	0.2	10^{-6}	0.1	None
**Al_2O_3**	**0.4**	**10^{-22}**	**0.3**	**Positive**
**CaO**	**0.5**	**10^{-51}**	**0.4**	**Positive**
**Na_2O**	**0.5**	**10^{-47}**	**0.3**	**Positive**
**K_2O**	**0.6**	**10^{-62}**	**0.3**	**Positive**
FeO_T	*-0.5*	*10^{-39}*	*-0.1*	*Negative*
MgO	*-0.5*	*10^{-44}*	*-0.1*	*Negative*

SO_3	-0.1	10^{-2}	0.1	None
Cl	0	10^{-1}	0	None
Quartier				
SiO_2	-0.1	10^{-1}	0.1	None
Al_2O_3	0.2	10^{-7}	0.3	None
CaO	0.2	10^{-9}	0.3	None
Na_2O	0.1	10^{-3}	0.2	None
K_2O	0.1	10^{-5}	0.4	None
FeO_T	0	10^{-1}	0	None
MgO	-0.2	10^{-7}	0	None
SO_3	-0.1	10^{-2}	0.1	None
Cl	0.2	10^{-8}	0.4	None

Fig. S10: Calculated Pearson's *r* index values for random permutations of each paired SHERLOC luminescence and PIXL P_2O_5 wt% image dataset. The leftmost column represents 100% permutation or completely randomized images. The rightmost column represents randomization of only 10% of the image for a sensitivity check of the method. *r* values of the measured data are written for each dataset for comparison. Note that *r* values of the measured data are well-above the distribution of index values from 100% and 10% permutation.

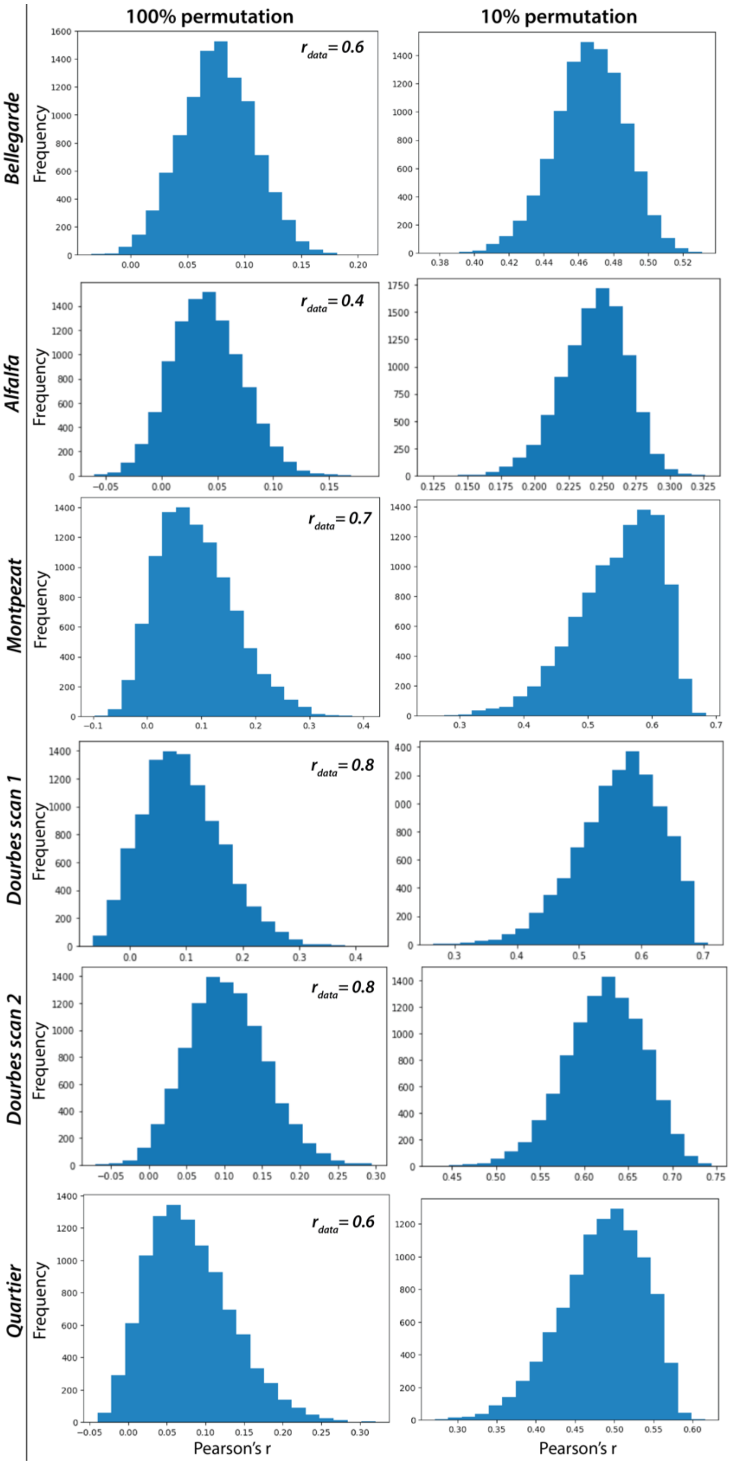

Fig. S11: Same as Fig. S10 but repeated for *SSIM* values. Note again that all *SSIM* values of the real data are well-above the distribution of 100% permutation, although this is not the case for some of the 10% permutation distributions.

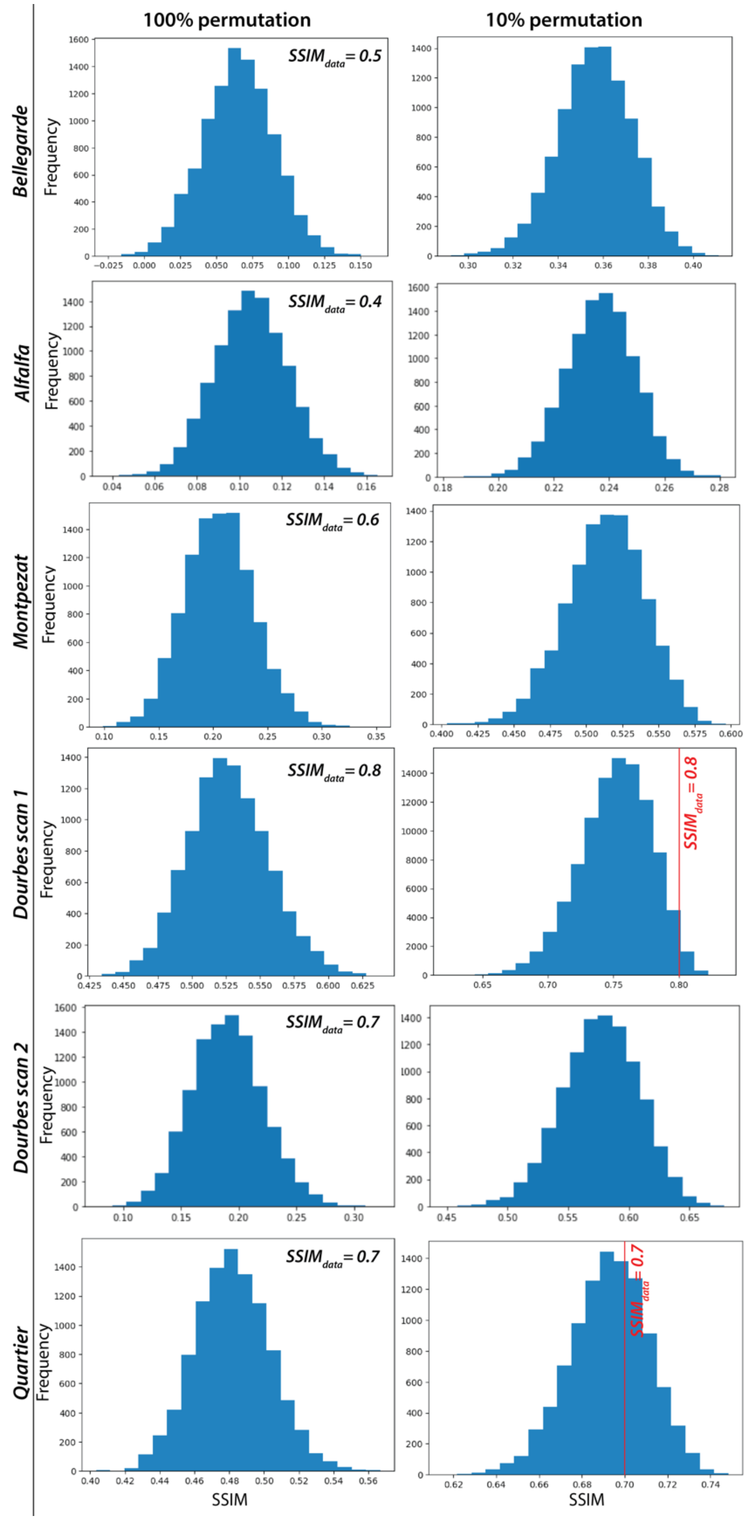

Table S3. Correlation statistics for 270-290 nm luminescence maps and all PIXL element maps. There is no clear indication of a statistically significant correlation between 270-290 nm luminescence as reflected in Fig. 8. However, a slightly positive and statistically significant *r* value can be found for SiO_2 in Bellegarde and for K_2O and SiO_2 in Alfalfa, which is predicted based on Fig. 8. *SSIM* values, however, do not indicate any enhanced correlation compared with correlations to other elements.

**Target**	***r*** **(lum.-SiO_2)**	***p-value*** **(lum.-SiO_2)**	***r*** **(lum.-K_2O)**	***p-value*** **(lum.-K_2O)**	***r*** **(lum.-element)**	***SSIM*** **(lum.-SiO_2)**	***SSIM*** **(lum.-K_2O)**	***SSIM*** **(lum.-element)**
Bellegarde	0.2	10^{-9}	0	0.3	0	0.3	0	0-0.3
Alfalfa	0.2	10^{-10}	0.2	10^{-9}	<0-0.1	0.2	0.2	0-0.1
All	0.2	10^{-20}	0.1	10^{-9}	0	N/A	N/A	N/A

Fig. S12: (A) Summary of SHERLOC 330-350 nm luminescence (stars) and phosphate Raman identifications (yellow circles) overlaid on PIXL P_2O_5 wt% abundance maps. These maps are also displayed in Figs. 6, S4, and S6. All 330-350 nm luminescence features that did not occur with a SHERLOC phosphate Raman detection and were not spatially in the vicinity of PIXL P_2O_5 detections are marked with red stars and an arrow. (B) Display of all spectra marked with a red star and arrow in panel A. (C) FWHM, wavelength position of max. intensity, and max. intensity of points associated with P_2O_5 and a phosphate Raman peak, and neither of those. The selection of points in the analysis here will be affected by uncertainties in the correlation algorithm and projection of X-ray points (Methods). Given that there is no distinguishing metrics observed for spectra here, this observation would extend to any spectra uncorrelated to phosphate Raman peaks and PIXL observed P_2O_5 missed due to uncertainties in correlation and X-ray point projection. This would be most applicable to the Alfalfa target (Methods).

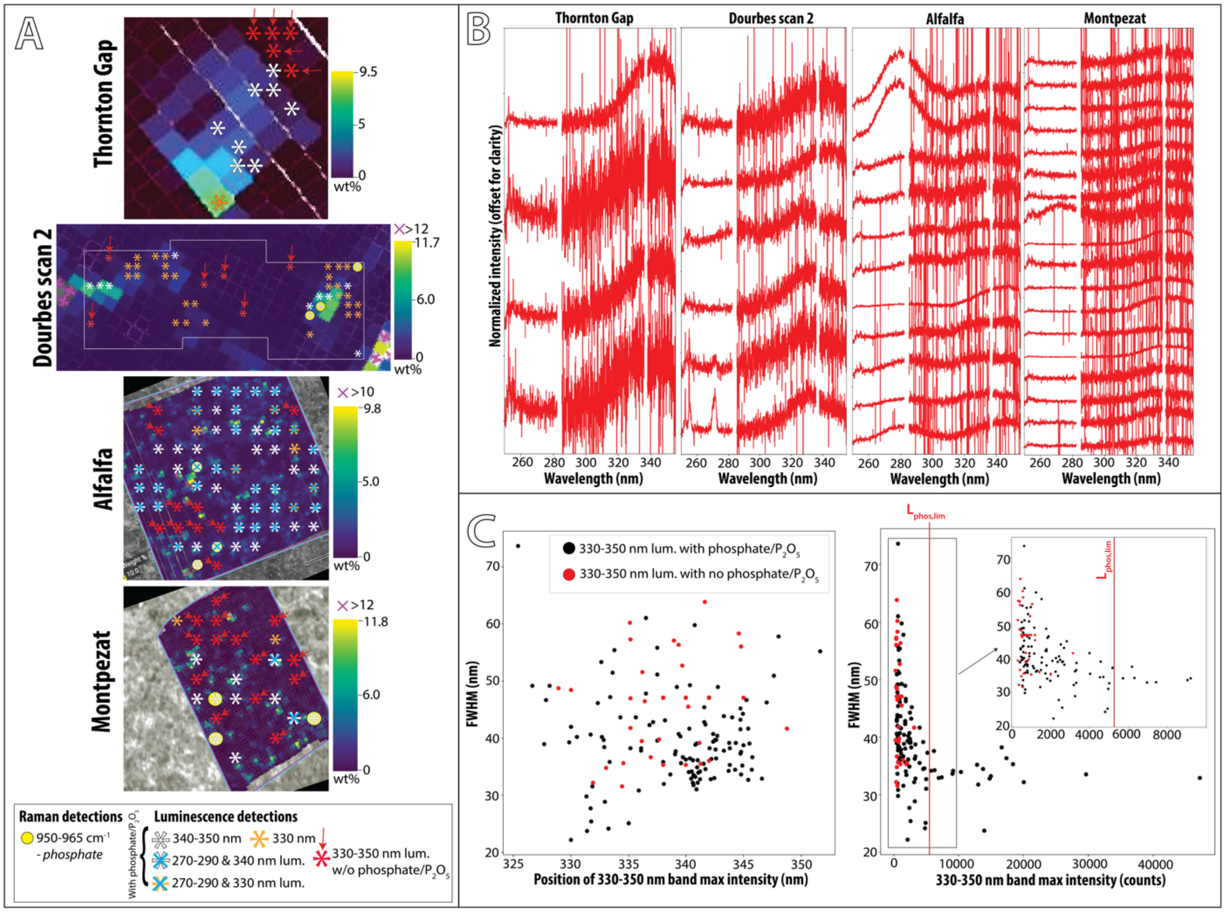

Fig. S13: Correlation plots showing max intensity and the wavelength position of max intensity for spectra where a 270-295 nm and a 330-350 nm band occur together in the same spectrum from HDR and detail scans of targets in the Máaz and Séítah fms. Note no observed correlation between 270-295 nm and 330-350 nm luminescence data. Note that Séítah fm targets, Dourbes and Garde, generally have a wavelength position of max intensity at ~290-295 nm, while spectra from the Máaz fm span from 270-295 nm. All targets contain spectra that span from 325-350 nm in association with the 270-295 nm luminescence bands. The Alfalfa target contains the most 270-295 luminescence (Table S1) and the most spectra with coinciding 270-290 nm and 330-350 nm luminescence bands.

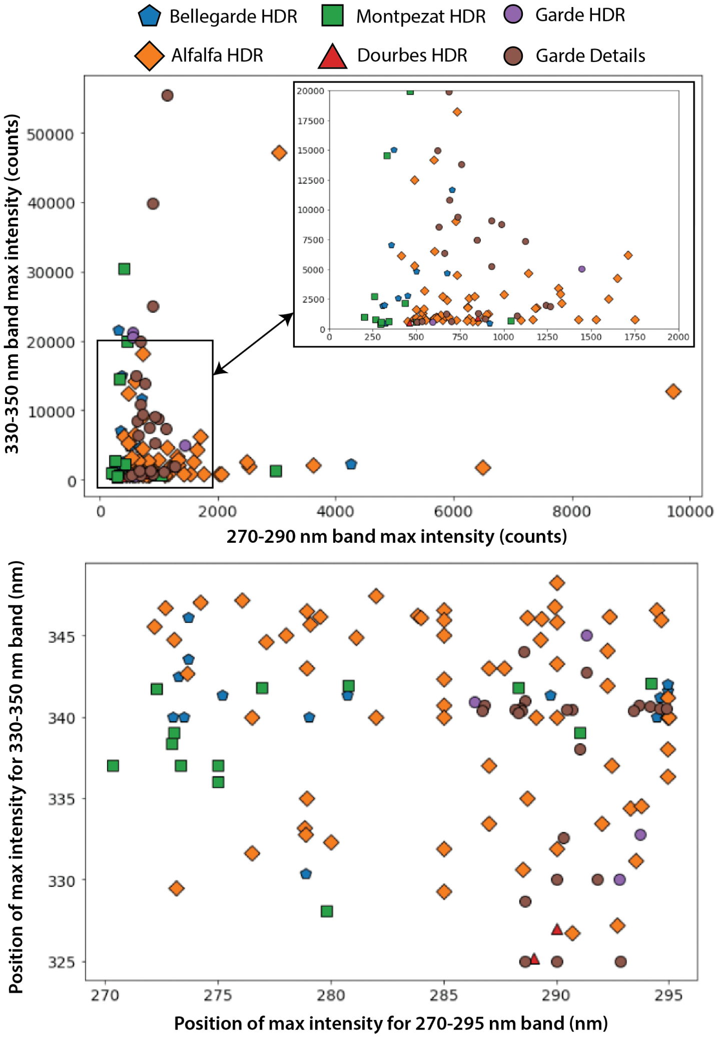

Supplement: Supplementary file 1 — Supplementary Text Figs. S1 to S13 Tables S1 to S3 [file sciadv.adm8241_sm.pdf]
